# Image-guided genomics of phenotypically heterogeneous populations reveals vascular signalling during symbiotic collective cancer invasion

**DOI:** 10.1038/ncomms15078

**Published:** 2017-05-12

**Authors:** J. Konen, E. Summerbell, B. Dwivedi, K. Galior, Y. Hou, L. Rusnak, A. Chen, J. Saltz, W. Zhou, L. H. Boise, P. Vertino, L. Cooper, K. Salaita, J. Kowalski, A. I. Marcus

**Affiliations:** 1 Graduate Program in Cancer Biology, Emory University, 1365C Clifton Road, Atlanta, Georgia 30322, USA; 2 Winship Cancer Institute, Emory University, 1365C Clifton Road, Atlanta, Georgia 30322, USA; 3 Department of Chemistry, Emory University, 506 Atwood Drive, Atlanta, Georgia 30322, USA; 4 Department of Biomedical Informatics, Emory University, 36 Eagle Row, Atlanta, Georgia 30322, USA; 5 Department of Biomedical Informatics, Stony Brook University, Stony Brook, New York 11794, USA; 6 Department of Hematology and Medical Oncology, Emory University, 1365C Clifton Road, Atlanta, Georgia 30322, USA; 7 Department of Radiation Oncology, Emory University, 1365C Clifton Road, Atlanta, Georgia 30322, USA; 8 Department of Biostatistics and Bioinformatics, Emory University, 1365C Clifton Road, Atlanta, Georgia 30322, USA

## Abstract

Phenotypic heterogeneity is widely observed in cancer cell populations. Here, to probe this heterogeneity, we developed an image-guided genomics technique termed spatiotemporal genomic and cellular analysis (SaGA) that allows for precise selection and amplification of living and rare cells. SaGA was used on collectively invading 3D cancer cell packs to create purified leader and follower cell lines. The leader cell cultures are phenotypically stable and highly invasive in contrast to follower cultures, which show phenotypic plasticity over time and minimally invade in a sheet-like pattern. Genomic and molecular interrogation reveals an atypical VEGF-based vasculogenesis signalling that facilitates recruitment of follower cells but not for leader cell motility itself, which instead utilizes focal adhesion kinase-fibronectin signalling. While leader cells provide an escape mechanism for followers, follower cells in turn provide leaders with increased growth and survival. These data support a symbiotic model of collective invasion where phenotypically distinct cell types cooperate to promote their escape.

## Abstract (web summary)

The mechanisms linking phenotypic heterogeneity to collective cancer invasion are unclear. Here the authors develop an image-guided genomic technique to select and amplify leader and follower cells from *in vitro* invading cell packs and find a cooperative symbiotic relationship between these two cell populations.

A single tumour can harbour distinct genetic and epigenetic cellular subpopulations that drive tumour initiation and progression. This intratumor heterogeneity is proposed to be one of the major confounding factors of treatment causing relapse and poor clinical outcome^1^. Genomic instability and epigenetic modifications generate intratumor heterogeneity^2^,^3^,^4^,^5^,^6^,^7^ creating distinct genetic and epigenetic subpopulations or clones^5^,^8^,^9^,^10^,^11^. A branched tumour evolutionary architecture can emerge^12^,^13^ containing the plasticity to progress under harsh environmental conditions and thwart therapeutic attempts to eradicate the tumour^2^,^8^. It can be argued that until we discover how intratumor heterogeneity can be circumvented, precision oncology initiatives may fall short of expectations^2^,^14^,^15^,^16^.

Single cell sequencing methodologies^17^,^18^,^19^ have improved the genomic, transcriptomic and epigenomic resolution of clonal tumour populations; however, the phenotypic implications of these alterations remain unclear. This is partly due to experimental challenges and is compounded by phenotypic plasticity that allows cancer cells to adapt to local changes in the microenvironment, without changes to the genome itself (for example, epithelial to mesenchymal transition^20^). Despite repeated observations that a small number of rare cancer cells or clones, hidden within a larger tumour population can drive tumour growth and spread^11^,^21^,^22^,^23^,^24^,^25^,^26^, studies linking single cell or clonal phenotypes with genomic data have been limited.

To probe the biology of a rare and phenotypically heterogeneous cell populations, single cells or subclones need to be isolated based upon user-defined criteria, instead of a random isolation approach; therefore, we developed a technique to image live cells within a biologically relevant three dimensional (3D) environment, select a cell or cellular group based upon user-defined criteria, extract the cell(s) and subject the cell(s) to genomic and molecular analyses. In this way, we can purify, amplify and systematically dissect the biologies of rare cells. This technique, termed spatiotemporal genomic and cellular analysis (SaGA), was used to dissect the phenotypic heterogeneity of collective cancer cell invasion in a 3D lung cancer model. These data incorporate the first SaGA-derived leader and follower cell lines to reveal that leader cells utilize atypical vasculogenesis signalling machinery by secreting vascular endothelial growth factor (VEGF) to attract follower cells in invasive cell chains. In contrast, follower cells support leader cell growth by increasing their mitotic efficiency. This relationship argues for a cellular symbiosis within the collective invasion pack. Furthermore, these data provide proof of concept that SaGA is a powerful technology for dissecting phenotypic heterogeneity within cancer cell populations.

## Results

### Leader cells are a unique and invasive subpopulation

**Figure 1:**
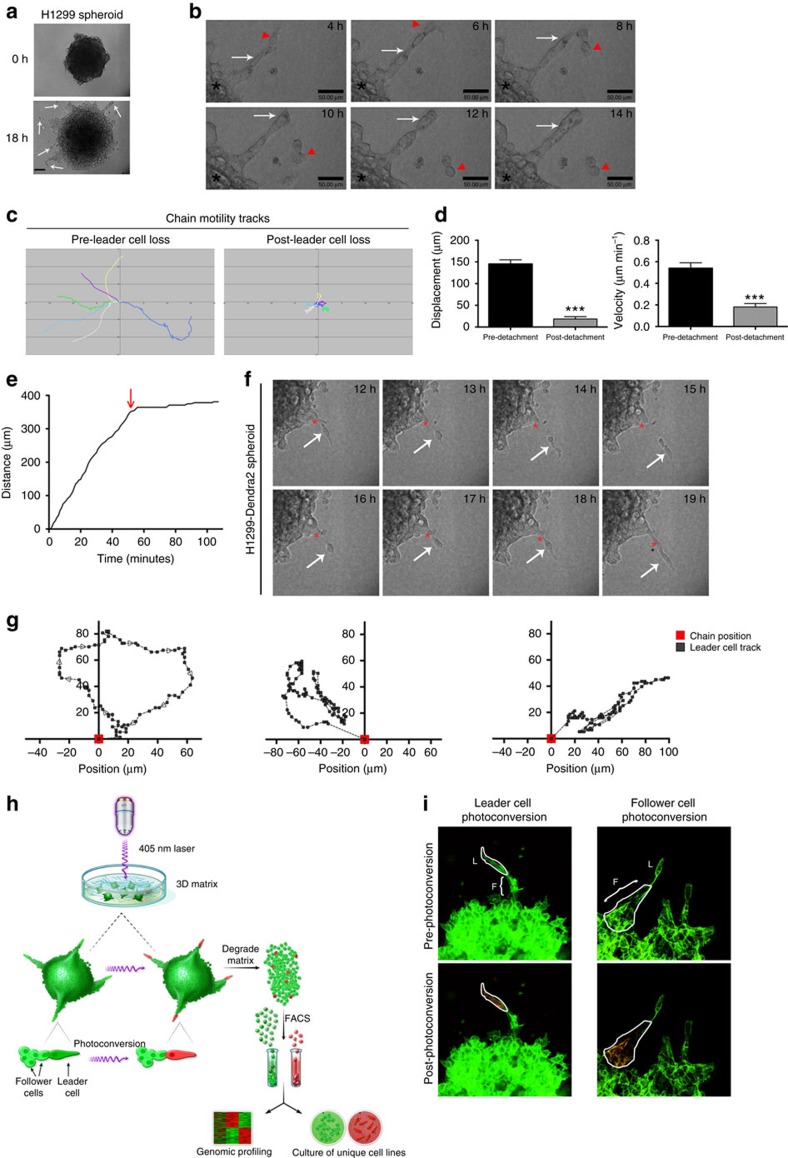
Leader cells represent a specialized subpopulation which can be purified using SaGA. (**a**) H1299 spheroids were imaged at 0 h (top) and 18 h (bottom) post embedding. Arrows, leader–follower invasive chains. (**b**–**f**) H1299 spheroids imaged using live cell confocal imaging. (**b**) Time lapse of H1299 spheroids acquired every 2 h. Arrow, follower cells in invasive chain; arrowhead, leader cell. (**c**) Invasive chains were tracked over time before (pre-leader cell loss) and after (post-leader cell loss) a leader cell becomes detached from the chain. Cell track plots are shown where each colour represents a single chain. (**d**) Quantification of track displacement and velocity of invasive chains from **c**. *n*=6. Error bars denote the s.e.m. ***P<0.001, using a Student's *t*-test. (**e**) Single invasive chain distance tracked over time. Arrow=point of leader cell detachment. (**f**) Representative still images of live cell imaging of H1299 spheroids taken every hour. Arrow denotes the leader cell, asterisk denotes the follower cell chain. (**g**) Three leader cells were tracked after the time of detachment from the rest of the chain. The position of each leader cell (grey track) is plotted relative to the position of the chain from which it detached (red point). (**h**) Schematic showing the process of the SaGA technique (see methodology for details). Printed with permission from Fairman Studios, LLC. This image is not included in the Creative Commons licence for the article. (**i**) Photoconversion examples using 3D spheroids of H1299-Dendra2 cells. F, follower cells; L, leader cell.

H1299 non-small cell lung cancer (NSCLC) tumour spheroids were embedded in a 3D matrix (Supplementary Fig. 1A,B) and imaged over time. Invading cells displayed phenotypically heterogeneous, collective chain invasion with leader cells defined as the first cell of a chain with trailing follower cells (Fig. 1a and Supplementary Movie 1). Upon leader cell detachment, the chain did not progress further (Fig. 1b), and this lack of plasticity was observed in 70% of all observed cases of leader cell detachment (Supplementary Fig. 1C). Quantification of invasive chain dynamics pre- and post-leader cell loss show that the distance travelled (Fig. 1c), displacement and chain velocity significantly decreased upon leader cell detachment (Fig. 1d). A single chain plot over time demonstrates that invasion distance plateaus after leader cell detachment (Fig. 1e), and once leader cells detach, they attempt to return to the chain (Fig. 1f,g). Similar findings were observed in a second NSCLC line, H1792 (Supplementary Movie 2); however, follower chain progression occurs post-leader cell detachment but in the direction of the leader cells, suggesting that the chain is attempting to re-attach (Supplementary Fig. 1D). Taken together, these data suggest that the leader cell is a specialized and essential cell type existing within a phenotypically heterogeneous cancer cell population.

### Development of image-guided genomics technology

To probe phenotypic heterogeneity, we developed a technique termed SaGA that allows for precise selection of user-defined living cells within a dynamic environment (Fig. 1h). H1299 or H1792 lung cancer cells were stably transfected with Dendra2, a photoconvertible fluorophore (Fig. 1h) with a plasma membrane-targeting palmitoylation tag^27^, allowing us to define individual cells during imaging. Before photoconversion, all cells have green fluorescence (Fig. 1h and Supplementary Fig. 2A, top) but upon user-defined exposure to 405 nm laser, only the selected cell is photoconverted to emit red fluorescence (Fig. 1h and Supplementary Fig. 2A, bottom). This process was tested in 3D models, where a single leader cell or group of follower cells were photoconverted without any measurable photoconversion in neighbouring cells (Fig. 1i) and without observable DNA damage (Supplementary Fig. 2B). SaGA steps were then optimized to specifically target, extract and amplify purified leader and follower cells from a 3D microenvironment.

### SaGA-derived leader cells maintain invasive potential

**Figure 2:**
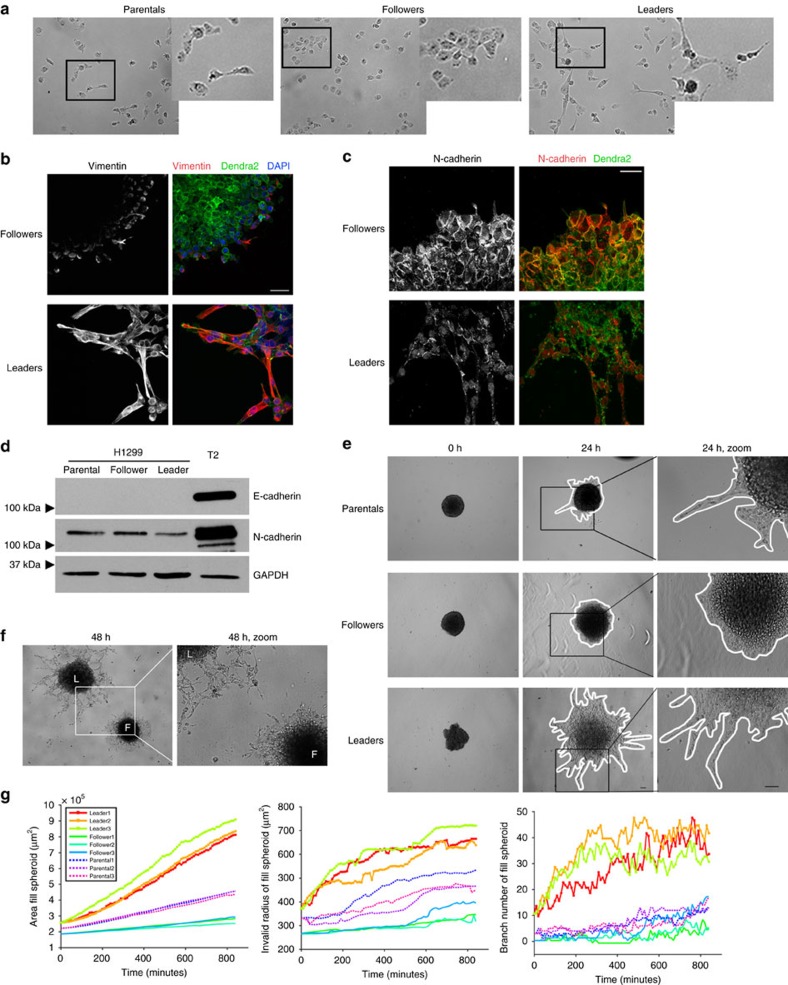
Leader cells maintain their invasive phenotype even while cultured as a purified population. (**a**) Images of H1299 follower and leader purified cells compared to the parental line. Zoomed images are shown to the right. (**b**) Immunofluorescence staining of vimentin (red) in follower and leader purified spheroids. Scale bar, 50 μm. (**c**) Immunofluorescence imaging of N-cadherin in purified leader and follower spheroids. Scale bar, 50 μm. (**d**) Western blot of H1299 parental, follower and leader cells for E-cadherin and N-cadherin. The T2 mouse tumor-derived cells are a positive control for E-cadherin. GAPDH as a loading control. (**e**) Spheroid invasion assays comparing H1299 parental, follower and leader spheroids. Zoomed images are on the right. Scale bar, 50 μm. (**f**) Leader (L) and follower (F) spheroids were embedded in proximity to each other in the same matrix. Images were taken at 48 h post embedding, with a zoomed image to the right. (**g**) H1299 parental, follower and leader spheroids were imaged using live cell confocal microscopy. Fluorescence images in both time and z were analysed for area, invasive radius and branch number. Three spheroids were analysed per condition.

SaGA was used to generate the first purified leader and follower cell lines from the parental line (H1299 or H1792 lung cancer cells) in 3D collectively invading spheroids. Single nucleotide polymorphism analysis verified that leader and follower lines created with SaGA originated from the H1299 parent line and not a contaminating cell type (Supplementary Table 1). Follower cells had an epithelial-like morphology in 2D culture, whereas leader cells were mesenchymal-like in shape (Fig. 2a and Supplementary Fig. 3A). Epithelial to mesenchymal transition (EMT) marker assessment showed that leader cells have increased staining of the mesenchymal protein vimentin in 3D spheroids compared to follower cells, which have little to no vimentin expression even in the few invasive cells (Fig. 2b). In contrast, the expression of the mesenchymal marker, N-cadherin, was decreased in leaders as compared to followers (Fig. 2c,d), suggesting a partial leader cell EMT. Both follower and leader cells are negative for the epithelial marker E-cadherin (Fig. 2d), consistent with H1299 cells. These data suggest that while leader cells have a more mesenchymal morphology and are vimentin positive, the traditional EMT signature alone cannot be utilized to identify leader cells.

The 3D invasive properties of SaGA-derived leader and follower cells were assessed and leader cell spheroids show significantly more invasion over time than follower and parental spheroids (Fig. 2e,g). Leader cell invasion resembles a network of interlinked cells when compared to sheet-like follower cell invasion (defined as invasion without the presence of leader–follower chains) (Fig. 2f and Supplementary Movies 3 and 4), as well as parental cells, which are more chain-like. Leader and follower invasion showed similar patterns in H1792-purified spheroids (Supplementary Fig. 3B). Quantitative analysis of spheroid invasive area and branch number over time show both are significantly increased in H1299 leader spheroids compared to follower spheroids (Fig. 2g). Leader cells maintain this invasive morphology and pattern in purified culture, whereas follower cells revert back to parental-type invasion after 1–2 months in culture similar to Fig. 1a.

### Leader cells promote leader–follower chain invasion

**Figure 3:**
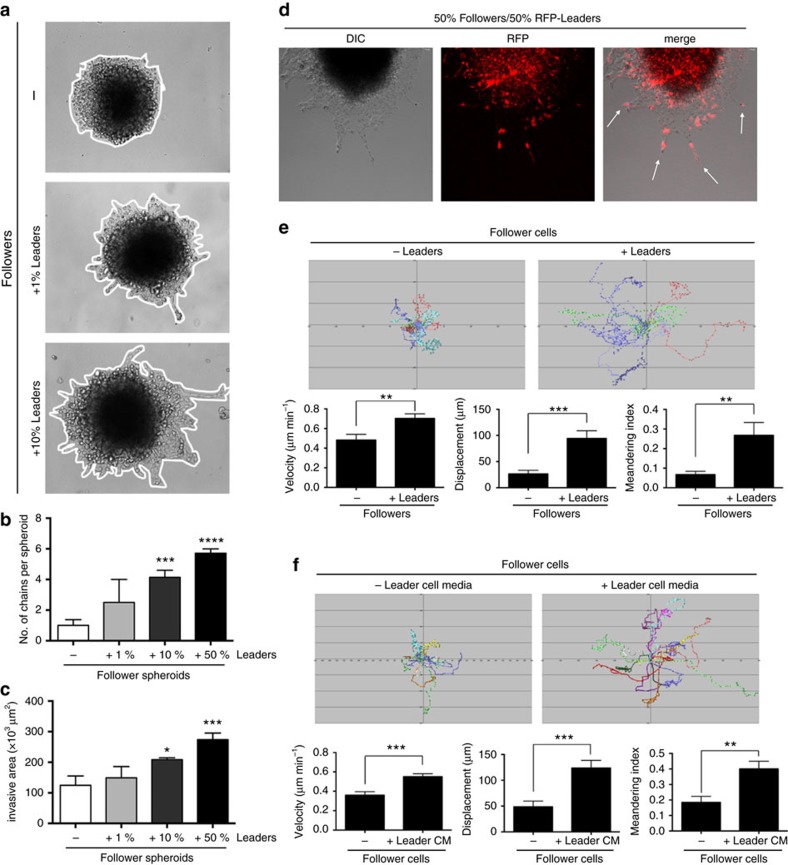
Leader cells promote motility and invasion of follower cells through a soluble factor. (**a**) Spheroids were formed from follower cells either alone (−) or in combination with increasing amounts of leader cells. (**b**) Quantification of the number of chains per spheroid in the experiment from **a**. *n*=6–7 spheroids. ***P<0.001, ****P<0.0001, using an ordinary one-way ANOVA with a Tukey's multiple comparisons test. (**c**) Quantification of invasive area from the experiment in **a**. *P<0.05, ***P<0.001. (**d**) Spheroid invasion assay with RFP-expressing leader cells mixed with follower cells at a 50:50 ratio in a spheroid invasion assay. RFP-leader-positive chains are denoted by white arrows. (**e**) Follower cells were plated in 2D either alone or in co-culture with RFP-leader cells. Single follower cells were tracked over time. Cell track plots as well as quantification of these tracks are shown. *n*=10–13 cells. **P<0.01, ***P<0.001, using a Student's *t*-test. (**f**) Follower cells were plated in 2D in either their own or in LCM. Follower cell tracks are plotted and quantified below. *n*=15–17 cells. **P<0.01, ***P<0.001 using a Student's *t*-test. Error bars denote the s.e.m.

To characterize leader–follower chains, leader cells were added to follower cell spheroids at 1, 10 and 50% of total cell content. Follower cells show little to no invasion (Fig. 3a), and what little invasion does occur is a sheet-like pattern as previously seen (Fig. 2f); however, adding 1% leader cells to the follower cell spheroid restored leader–follower chains that are morphologically similar to the parental line, and this trend continued in a leader cell dose-dependent manner (Fig. 3a). The addition of 10 or 50% leader cells significantly increased the chain number and total invasive area (Fig. 3b,c). To test if leader cells retain leadership in these mixing experiments, red fluorescent protein (RFP)-expressing leader cells were made to track leader cells. RFP-leader cells are found at the leading tip of invasive chains 85% of the time (Fig. 3d). Leader cells were rarely observed in a non-leading position within the invasive chain, even when leader cells comprise 50% of the entire population, suggesting either that follower cells are selected for or leader cells are excluded from non-leading positions.

We analysed if leader cells could promote follower cell motility using 2D motility assays, since single cell motility and cell–cell interactions could be more easily visualized. Co-culture experiments were performed by mixing follower cells with RFP-leader cells, and cell motility was quantitatively assessed. Follower cells plated alone show active lamellipodia dynamics (Supplementary Movie 5) but had limited net movement (Fig. 3e). Adding ∼30% leader cells to the follower cell population significantly increased follower cell motility (Fig. 3e and Supplementary Movie 6). A similar set of experiments was performed to determine if this increased follower cell motility is due to leader cells themselves or a factor secreted by leader cells. Conditioned media from leader cells was sufficient to promote the motility of follower cells (Fig. 3f). Together, these data indicate that leader cells can stimulate the motility of follower cells via a secreted factor.

### Leader–follower invasion utilizes vascular signalling

**Figure 4:**
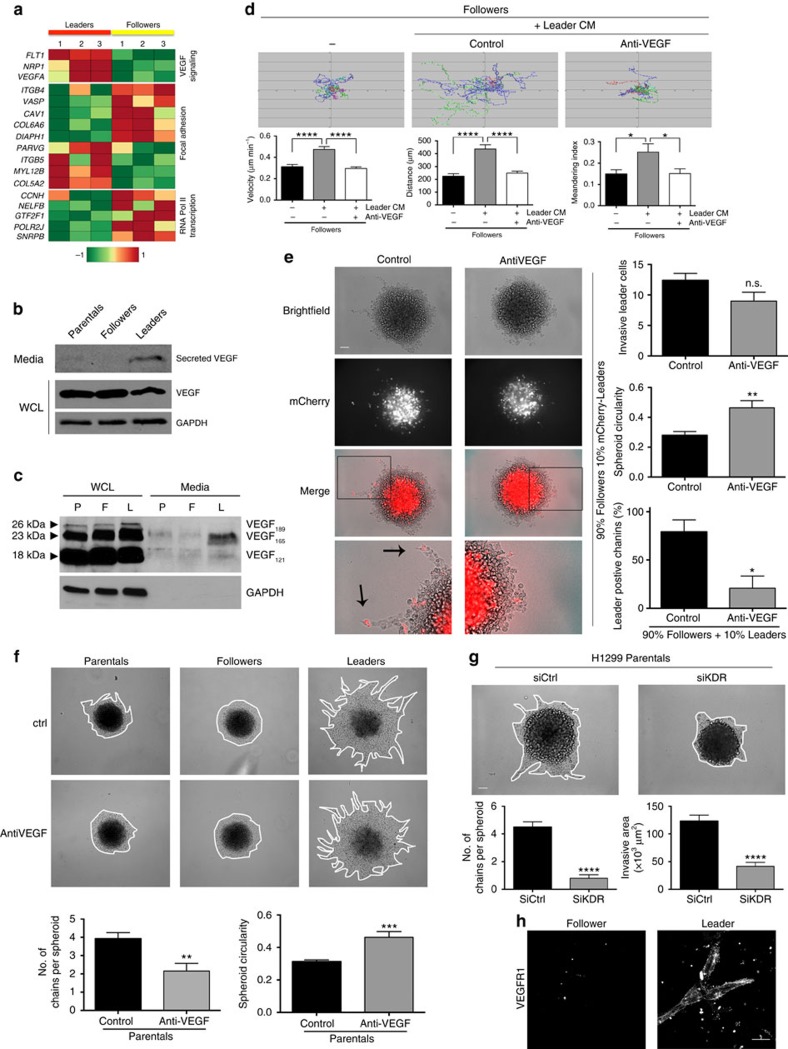
Gene and protein expression analyses reveal the VEGF pathway as enriched in leader cells. (**a**) Unsupervised hierarchical clustering of differentially expressed genes involved in three major pathways: VEGF, focal adhesion and RNA Polymerase II transcriptional regulation. The leader and follower experimental replicates are coloured in red and yellow, respectively. The hierarchical clustering was performed using Pearson correlation distance measure and average linkage method. (**b**) Western blot showing VEGF expression in media and whole-cell lysate (WCL) samples from H1299 parental, follower, and leader cells. GAPDH was used as a loading control. (**c**) Western blot of various VEGF isoforms in H1299 parental (P), follower (F), and leader (L) WCLs and media samples. GAPDH was as a loading control. (**d**) Live cell tracking analysis of follower cells plated in 2D either alone (−), in leader conditioned media (CM) or leader CM pre-treated with a VEGF-neutralizing antibody (anti-VEGF). Cell track plots from each condition are shown, and bar graphs from these tracks are below. *n*=18 cells. *P<0.01, ****P<0.0001 using an ordinary one-way ANOVA with a Tukey's multiple comparisons test. (**e**) The VEGF-neutralizing antibody in 3D spheroid invasion assays. Mixed spheroids were generated using 90% follower cells, 10% RFP-positive leader cells. Zoomed images are shown below. Arrows denote RFP-leader-positive invasive chains. The total number of invasive leader cells, the spheroid circularity as an indirect measure of sheet-like invasion, and the percentage of leader-positive chains in each condition were quantified and graphed to the right. Scale bar, 100 μm. *P<0.05, **P<0.01, using Student's *t*-tests. (**f**) Spheroid invasion assay of H1299 parental, follower and leader spheroids in the presence of the anti-VEGF antibody as compared to vehicle control. The number of chains per spheroid and spheroid circularity for the parental spheroids is graphed below. *n*=12–13 spheroids. **P<0.01, ***P<0.001. (**g**) Spheroid invasion assay on H1299 parentals treated with either VEGFR2 (KDR) siRNA or scrambled siRNA control. The number of chains/spheroid and invasive area are graphed below. *n*=10 spheroids. ****P<0.0001. (**h**) Immunofluorescence imaging of VEGFR1 in follower and leader spheroids. Scale bar, 20 μm. Error bars denote the s.e.m.

To probe the underlying biological mechanisms that drive leader and follower cell biology and communication, transcriptome profiling was performed. There were 788 candidate transcripts that were upregulated in leader cells compared to follower cells (Supplementary Fig. 4A) and 684 transcripts upregulated in follower cells compared to leader cells (Supplementary Fig. 4B). These results incorporate controls for the SaGA photoconversion approach (see methodology) and therefore all significant transcripts in leaders versus follower must not have significantly changed between control samples (Supplementary Fig. 4C). Functional interaction networks revealed several significant networks related to VEGF, focal adhesion signalling and RNA Pol II transcription (Fig. 4a and Supplementary Fig. 4D) that vary in leader cells compared to follower cells. Specifically, VEGF signalling transcripts were significantly increased in leader cells compared to follower cells, whereas focal adhesion signalling transcripts were more heterogeneous with some significantly increased or decreased in leader compared to follower cells.

VEGF signalling was particularly interesting since morphologic patterning of endothelial cells during vascular sprouting has leader-like tip cells with follower-like stalk cells, and Fig. 3f supports the concept of a secreted factor stimulating follower cell movement. VEGF secretion was found to be upregulated in leader cells compared to parental and follower cells (Fig. 4b and Supplementary Figs 5A and 9) with the VEGF_165_ isoform the most abundant (Fig. 4c and Supplementary Fig. 9). The addition of recombinant VEGFA to follower cells was sufficient to promote their motility in 2D (Supplementary Fig. 5B); however, VEGFA addition was not able to stimulate follower invasion in 3D (Supplementary Fig. 5C), suggesting that the leader cells themselves are necessary to promote follower invasion. Next, we probed if blocking VEGF signalling could impact leader cell influence on follower motility and invasion. An inhibitory anti-VEGF antibody was added to leader cell conditioned media that bathed 2D follower cell cultures, which was to sufficient to inhibit leader cell stimulation of follower cell motility (Fig. 4d). To determine if this is observed during 3D collective invasion, the anti-VEGF antibody was added to mixed spheroids with 90% followers and 10% mCherry-leaders. In control-treated cells, mCherry-leader cells were observed at the tip of 80% of invasive chains (Fig. 4e); however, anti-VEGF treatment abolishes chain formation, and the percentage of chains positive for mCherry-leaders decreased to 20% (Fig. 4e). Chain invasion was significantly reduced in parental spheroids with anti-VEGF treatment, knockdown of *VEGFR2* (*KDR)* or treatment with the VEGFR2 kinase inhibitor, ZM323881 (Fig. 4f,g and Supplementary Fig. 5E–G). However, leader cell invasion itself was not dependent on VEGF signalling, as the total number of leader cells found in the entire invasive area (that is, independent of location within the invasive area) was not significantly reduced in the mixed spheroids (Fig. 4e). Additionally, purified leader spheroids remained highly invasive with anti-VEGF treatment (Fig. 4f). We hypothesized this may be due to expression of VEGFR1 decoy receptor, since leader cells had increased levels of VEGFR1 mRNA (*Flt1*; Fig. 4a and Supplementary Fig. 4D). Leader cells have significantly more VEGFR1 expression when compared to follower cells (Fig. 4h and Supplementary Fig. 5D), supporting the concept that VEGFR1 decoy receptor could dampen VEGF autocrine signalling in leader cells.

We probed other mechanisms controlling tip-stalk cell maintenance during vasculogenesis and found that VE-cadherin, the major cadherin that links endothelial cells during vasculogenesis, is highly expressed in leader cells but not in follower cells (Supplementary Figs 6A,B and 9), suggesting that leader–follower cell:cell contacts are maintained by VE-cadherin but follower–follower cell:cell contacts are not. We also focused on canonical Notch-Dll4 signalling^28^ during collective invasion. Notch protein is highly expressed in follower cells but not in leader cells, while Dll4 is expressed in leader but not follower cells (Supplementary Fig. 6C–E). This mimics tip-stalk cell expression patterning during vasculogenesis, further supporting the concept of a vascular mimicry during collective cell invasion. To probe if Notch1 acts as a leader cell-suppressing signal, similar to its role in regulating tip cell formation in vasculogenesis^28^,^29^, we utilized the γ-secretase inhibitor RO4929097, which inhibits Notch1 activity (Supplementary Figs 6F and 9). Surprisingly, inhibition of Notch1 signalling via RO4929097 treatment did not promote the leader cell phenotype as observed in other models^30^ but instead, blocked chain invasion in H1299 parental spheroids (Supplementary Fig. 6G). We hypothesized that the role of Notch1 in follower cells in our system must differ from its canonical role in tip cell formation and we therefore tested whether follower cells may utilize Notch signalling to promote proliferation. This result showed that treatment with RO4929097 significantly decreased follower cell growth (Supplementary Fig. 6H). These data taken together suggest that leader–follower cells utilize a non-canonical vascular signalling program to promote formation of the invasion chain.

### Fibronectin-FAK signalling drives leader cell invasion

**Figure 5:**
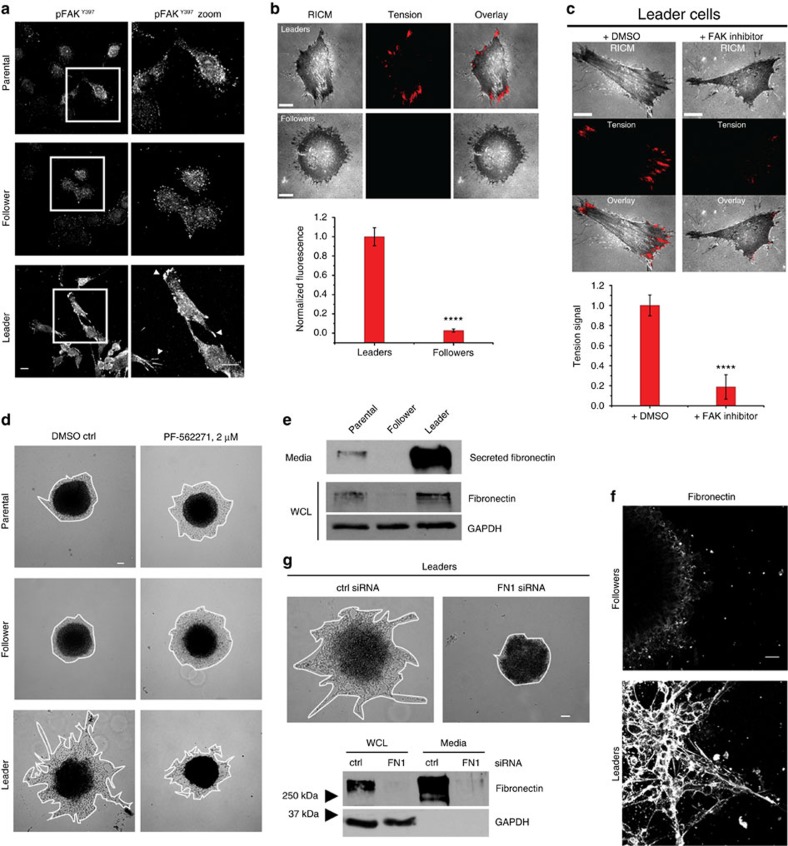
The fibronectin/FAK pathway drives leader cell invasion. (**a**) Immunofluorescence imaging of pFAK^Y397^ in H1299 parental, follower and leader cells plated in 2D. Arrowheads denote large adhesion sites in leader cells. Scale bar, 20 μm. (**b**) Representative reflection interference contrast microscopy (RICM) and integrin tension images of leader and follower cells^32^. An overlay of the RICM and integrin tension channels demonstrates that regions at the cell edge associated with FAs generate sufficient tension to unfold the titin-based probe for the leader cells. Plot below shows the average integrin tension signal for leaders and followers. Error bars represent the s.e.m. from 10 cells for each group collected from three chambers, ****P<0.0001 using a Student's *t*-test. Scale bars, 10 μm. (**c**) Representative RICM and integrin tension images for leader cells incubated in the presence of either 0.1% DMSO control and PF-562271 FAK inhibitor. Error bars represent the s.e.m. from 10 cells for each group collected from three chambers, ****P<0.0001 using a Student's *t*-test. Scale bars, 10 μm. (**d**) Spheroid invasion assay with H1299 parental, follower or leader cells in the presence of 2 μM PF-562271 FAK inhibitor or DMSO control. Scale bar, 100 μm. (**e**) Western blot of fibronectin protein in the media or WCL of H1299 parental, follower and leader cells. GAPDH was a loading control. (**f**) Immunofluorescence of extracellular fibronectin in follower and leader spheroids. Scale bar, 50 μm. (**g**) Fibronectin (*FN1*) was knocked down in leader cells using targeted siRNA. Scrambled siRNA was used as a negative control. Spheroids from *FN1* siRNA-treated leader cells were assayed for invasion compared to control. Western blot below confirms knockdown efficiency. Scale bar, 100 μm.

Transcriptome data showed significant changes in cell adhesion pathways between leader and follower cells; therefore, activated focal adhesion kinase (FAK) was assessed in pure leader cells compared to parental and follower cells. Leader cells show increased pFAK^Y397^ at the leading edge compared to followers (Fig. 5a) with larger and more elongated adhesion sites. We reasoned that enlarged pFAK-positive adhesion sites may help leader cells generate traction force during migration. To test this, leaders were plated onto a glass slide decorated with molecular tension probes that quantitatively map integrin forces using fluorescence microscopy^31^ (Supplementary Fig. 7A). Two hours after plating on the sensor, leader cells showed a positive tension signal at the sites associated with focal adhesions, thus indicating integrin-ligand forces that exceed 36 pN shortly after adhering^32^ (Fig. 5b). In contrast, follower cells do not generate sufficient tension to unfold the probe within this time frame. To determine if this enhanced integrin force is mediated through FAK and whether it is important for 3D invasion, leader cell spheroids were treated with the FAK inhibitor, PF-562271. As compared to DMSO control, leader cells treated with FAK inhibitor had significantly reduced integrin force as well as reduced spheroid invasion (Fig. 5c,d). Interestingly, FAK inhibition in the follower cells promoted sheet-like invasion (Fig. 5d), suggesting differing functions of FAK in leader and follower populations.

To probe FAK-based signalling in leader cells, fibronectin was assessed since it is a major ligand for the integrin-FAK pathway. Leader cells had significantly more cellular and secreted fibronectin than parental and follower cells (Fig. 5e and Supplementary Figs 5H and 9). Leader spheroids had a vast 3D fibronectin network but nearly no fibronectin was observed in follower spheroids (Fig. 5f and Supplementary Fig. 5I). Fibronectin siRNA depletion abolished leader cell invasion (Fig. 5g and Supplementary Figs 7B and 9), similar to pharmacological FAK inhibition, showing that this pathway is necessary for leader cell movement. Additionally, depleting FAK in leader cells before mixing in spheroids with follower cells reduces the number of leader-positive chains per spheroid (Supplementary Figs 7C and 9), supporting the concept that FAK is required for leader cells to generate invasive chain motility. Taken together, these data show that leader cells depend upon fibronectin-FAK adhesion signalling to create force during invasion into the microenvironment.

### Follower cells are proliferative and rescue leader defects

**Figure 6:**
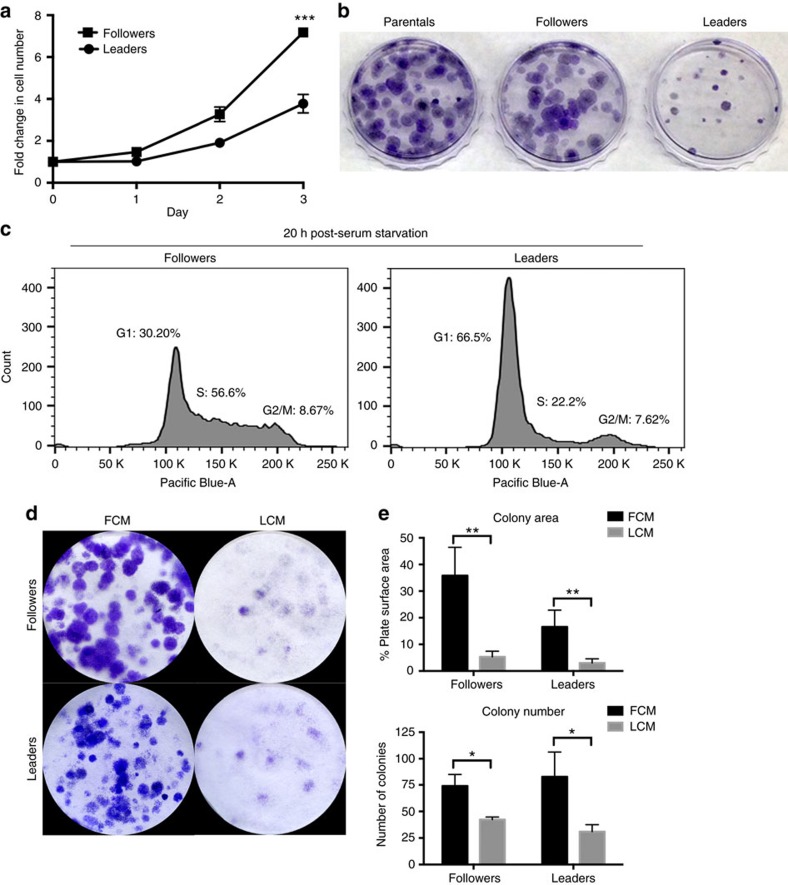
Follower cells are a proliferative subpopulation promoting leader cell growth via a secreted factor. (**a**) Graph showing H1299 follower and leader cell growth over 3 days. ***P<0.001. (**b**) Colony formation assay of H1299 parental, follower and leader cells. (**c**) Cell cycle analysis of H1299 follower and leader cells that were serum starved, and then released for 20 h with normal growth media. (**d**) Colony formation assay of H1299 follower and leader cells. Cells were plated in conditioned media from FCM or LCM. Images taken 2 weeks after plating. (**e**) The colony size and number of colonies from **d** were measured using ImageJ software. *P<0.05, **P<0.01 using a Student's *t*-test. Error bars denote the s.e.m.

The question remained as to why leader cells invade with follower cells, since purified leader cells are fully competent to invade alone. We hypothesized that follower cells may provide a benefit to invading leader cells. During the initial collection and expansion of leader and follower clones (Fig. 2a), we observed that leader cells grew at slower rates compared to follower cells; therefore, to test if follower cells are more proliferative, a basic proliferation assay was performed, showing that follower cells had increased cell counts after 3 days compared to leader cells (Fig. 6a and Supplementary Fig. 8A). Similarly, colony formation assays showed that leader cells have little colony growth over time, whereas followers have a greater number of large colonies (Fig. 6b and Supplementary Fig. 8B). Cell cycle analysis 20 h post-serum starvation showed a large G1 population in leader cells compared to follower cells (Fig. 6c and Supplementary Fig. 8C,D); however, without serum starvation there are no differences in the cell cycle between these two populations (Supplementary Fig. 8E).

To test the hypothesis that followers provide a growth or survival advantage to leader cells, leader cells were subjected to a colony formation assay in the presence of leader cell conditioned media (LCM) or follower cell conditioned media (FCM). Leader cells in LCM had low colony formation as measured by colony number and area (Fig. 6d,e); in contrast, adding FCM to leaders resulted in a significantly increased colony number and area. Strikingly, the addition of LCM to follower cells significantly inhibited colony growth as compared to followers grown in their own conditioned media (Fig. 6d,e). Taken together, these data show that FCM can significantly increase colony growth of leader cells, whereas LCM inhibits colony growth of follower cells.

**Figure 7:**
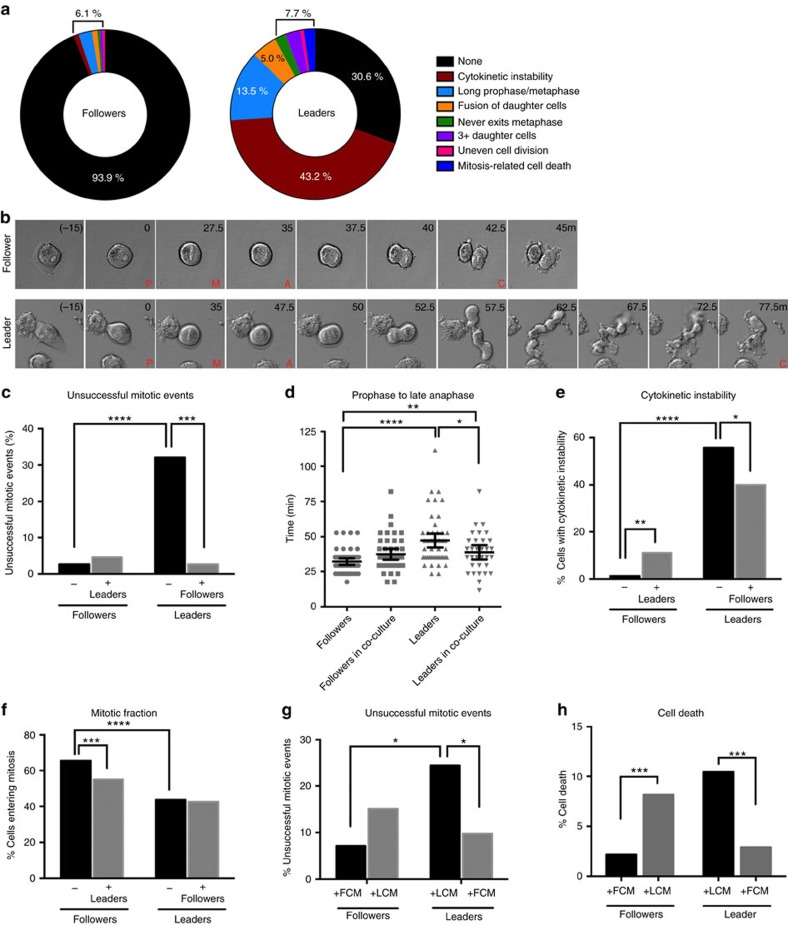
Mitotic defects observed in leader cells are rescued by the addition of follower cells. (**a**–**f**) H1299 follower and RFP-leader cells were plated in 2D alone or in a 50:50 mixed co-culture then imaged using live cell confocal microscopy. Mitotic events were analysed in each condition. (**a**) Graphs of each type of mitotic error noted during the live cell imaging as a percentage of all mitotic events. *n*=258. (**b**) Still images of a mitotic event in a follower cell and a leader cell. Time in minutes. P, prophase; M, metaphase; A, anaphase; C, cytokinesis. (**c**) Unsuccessful mitotic events were classified and graphed as a percentage of total mitotic events seen in followers, leaders or each cell type in the co-culture condition. *n*=506. A two-tailed *χ*^2^-test was used to determine significance. (**d**) Dot plot of the amount of time each cell spent from prophase to the beginning of anaphase. *n*=555 cells. A one-way ANOVA with Tukey's multiple comparisons test was used. Bars represent the median and 95% confidence intervals from 40 randomly selected cells. (**e**) Cytokinetic instability events were graphed as a percentage of all dividing cells. A two-tailed *χ*^2^- analysis was used. *n*=486. (**f**) Bar graph of mitotic fraction, defined as the number of cells that undergo mitosis as a percentage of total cells in the field of view. A two-tailed *χ*^2^- analysis was used. *n*=1,106. (**g**) Unsuccessful mitotic events were counted and graphed from conditioned media from FCM or LCM. A two-tailed *χ*^2^- analysis was used. *n*=185. (**h**) Leader and follower cells were cultured in LCM or FCM and cell death events were graphed as a percentage of total cells in the field of view. A two-tailed *χ*^2^- analysis was used. *n*=1782. *P<0.05, **P<0.01 ***P<0.001, ****P<0.0001.

To probe why leader cells have growth defects, live cell imaging was used to assess mitosis in purified populations. Leader cells had a variety of mitotic defects compared to follower cells (Fig. 7a), with the most prominent being cytokinetic instability (defined as initially having >2 daughter cells with excessive membrane blebbing and cell shape deformation during cytokinesis but over time corrected to two daughter cells; Fig. 7b). Other defects include increased time from prophase through anaphase and eventual fusion of daughter cells. Overall, ∼70% of all leader cells have mitotic defects, as compared to only 6% in follower cells (Fig. 7c).

To test if leader cell mitotic defects could be rescued by follower cells, follower cells were co-cultured with RFP-leader cells, and leader cell mitotic defects were nearly abolished (Fig. 7c). Co-culture with follower cells specifically rescued the prophase-to-anaphase mitotic delay observed in leader cells (Fig. 7d) and cytokinetic instability (Fig. 7e); however, the mitotic fraction of leader cells, defined as the percentage of cells entering mitosis in a field of view, was not impacted by follower cells (Fig. 7f). A similar effect on mitotic defects was observed using FCM on leader cell cultures where unsuccessful mitotic defects was significantly decreased as compared to leaders cultured in LCM (Fig. 7g). In addition to effects on mitotic efficiency, FCM also impacted the percentage of cells undergoing cell death. The addition of FCM to leader cells significantly reduced cell death as compared to leaders cultured in LCM (Fig. 7h). Conversely, LCM had the opposite impact on follower cells and increased cell death (Fig. 7h). Interestingly, follower cells when co-cultured with leader cells also have an increase in cytokinetic instability as well as a decrease in the overall mitotic fraction (Fig. 7e,f), suggesting that leader cells may hinder follower cell growth. Taken together, these data support a model whereby follower cells decrease mitotic defects and leader cell death while supporting leader cell colony formation, whereas leader cells increase these defects in follower cells thereby hindering follower cell growth.

## Discussion

SaGA combines microscopy, cell biology and genomics to isolate and amplify phenotypically distinct subpopulations within a larger, heterogeneous population. This technique lies at an emerging intersection of cell biology and genomics by combining single cell analysis with unbiased genomic datasets. We propose that SaGA can be used to isolate selected cells from phenotypically heterogeneous populations, including highly proliferative cells, drug-resistant cells or other microscopy-amenable phenotypes. Furthermore, since a protein can be Dendra2-tagged (as opposed to palmitoylation-tagged Dendra2 used here), subcellular localization could also be a stratifying phenotype to select cells with specific protein localizations.

We used SaGA here to probe the biological mechanisms that guide the phenotypic heterogeneity found in collectively cancer invasion (reviewed in refs 33, 34). The leader cell phenotype is stable over many generations of culture and maintains an invasive and networked morphology when compared to follower or parental cells (Fig. 2); therefore, leader cells are a stable phenotype and lack the phenotypic plasticity to revert back to a non-invasive phenotype. We do not observe follower cells taking on a leader cell position during collective invasion, which is in contrast to 2D wounding studies where leader cells are replaced^35^. In our studies, follower cells revert back to the parental phenotype (that is, gain collective invasion chains) suggesting that follower cells have greater phenotypic plasticity. We propose that leader cells are a specialized invasive cellular population, where phenotypic heterogeneity could be hardwired into their genome or epigenome. This is supported by previous studies that have found a specialized keratin-14 positive subpopulation capable of pioneering collective invasion in breast cancer^36^. Additionally, recent work shows that a distinct epigenetic state of a breast tumour cellular subpopulation promotes a transition to a more invasive cooperative cell invasion phenotype where canonical mesenchymal markers were insufficient to mark leader cells^37^. Similarly, leader cells here also lack a complete EMT signature (Fig. 2), supporting the concept that invasive cancer cells can have partial EMT phenotypes that generate phenotypic plasticity^38^.

Our transcriptomic data of SaGA-derived cell populations show several significant pathways enriched in leader or follower cells, including VEGF and adhesion signalling (Fig. 4). VEGF signalling was an attractive candidate since leader–follower collective invasion morphologically resembles VEGF-driven vascular sprouting^39^, which utilizes a leader-like tip cell and follower stalk cells. Our data support a model that resembles, but is not identical to, vascular sprouting. Leader cells secrete VEGFA, which is necessary for collective invasion pack formation, but not leader cell motility itself (Fig. 4). Importantly, this differs from the endothelial cell vascular sprouting where a tip cell chemotaxes along a VEGF gradient through a hypoxic microenvironment. The leader–follower chain mimics the expression pattern of canonical Notch-Dll4 endothelial cell expression patterns^28^, further supporting the concept of a vascular signalling mimicry; however, Notch1 expression in follower cells does not appear to repress the leader phenotype, as is observed in canonical stalk cell maintenance^28^,^29^. Instead, in our case, inhibiting Notch1 reduces collective chain invasion (Supplementary Fig. 6), suggesting an atypical vascular signalling pathway. Other reports have described a tumour cell vascular mimicry where cancer cells upregulate endothelial-like gene expression programs to form vessel-like structures that act as a functioning blood supply^40^,^41^. Vessel-like structures were not observed here but this possibility cannot be ruled out *in vivo*. Interestingly, while VEGFA is required for pack formation, the actual motility of leader cells is instead dependent upon the traction force generated by fibronectin-FAK signalling (Fig. 5). Fibronectin itself has been classically linked to cell invasion^42^,^43^, and leader cells secrete fibronectin at significantly higher levels than followers, and fibronectin is required for leader cell motility (Fig. 5 and Supplementary Fig. 5). Taken together, this supports a model where formation of the collective invasion pack is VEGF dependent and utilizes vascular signalling components, but motility itself requires fibronectin-FAK signalling.

**Figure 8:**
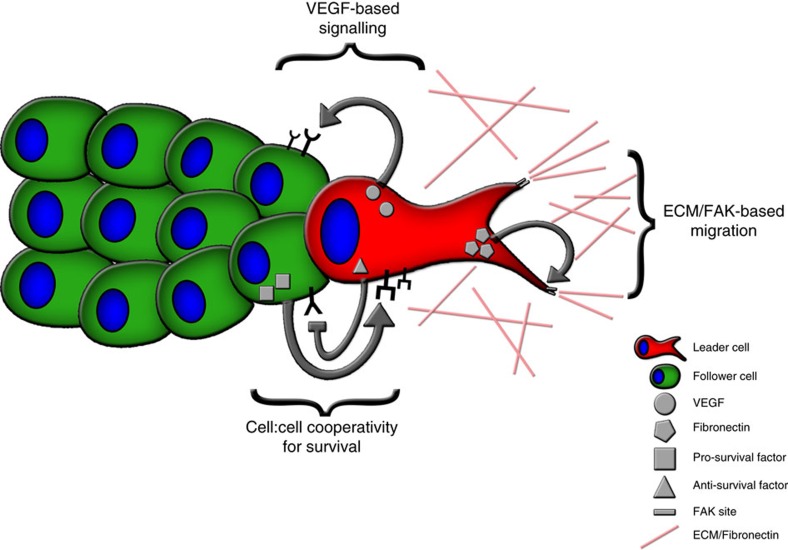
Model of cell symbiosis during collective cancer cell invasion. A collective invasion pack shows phenotypic heterogeneity consisting of at least two subpopulations, a highly invasive leader cell (red) at the front of the pack and follower cells (green) immediately attached to and following the leader cell. The leader cell has increased VEGFA secretion, which promotes the motility and invasion of the follower cells. Other vasculogenesis signalling molecules are also utilized in the cell:cell communication between leader and follower cells, including VEGFR1, VEGFR2, VE-cadherin and Notch1 (not shown in the model for brevity); This atypical VEGF-based signalling allows for successful chain formation during invasion. Concomitantly, leader cells secrete excess fibronectin, which activates the canonical integrin/FAK pathway. This pathway allows for leader cells to create force to move the invasive pack forward into the microenvironment. Conversely, follower cells are a highly proliferative population that promote leader cell growth via a secreted factor, whereas leader cells secrete a factor that hinders follower cell growth. These data support a symbiotic relationship in the collective invasion pack in which the follower cells promote leader cell survival and leader cells promote follower cell escape.

One important question is why do cancer cells invade as a collective pack as opposed to single cells? One potential answer is that the multi-cellular pack provides a survival or invasive advantage to escaping cells. In circulating tumour cells, cells that invaded as groups had greater success and worse clinical outcomes^44^,^45^. Furthermore, studies show that tumour cell clusters rather than single cells seed polyclonal metastases in mouse models^44^,^46^,^47^,^48^, supporting the concept of collective invasion and/or metastasis. We observe that leader cells are competent to invade, even when follower cells are absent; however, when co-cultured with followers, leader cells almost always invade with follower cells, suggesting that pack migration is preferred to single cell migration (Fig. 2). We show that followers provide a growth advantage to leader cells by increasing leader cell colony formation (Fig. 6 and Supplementary Fig. 8) and correcting their mitotic defects (Fig. 7). These data argue for a symbiotic relationship between leader and follower cells, where the follower cell secretome improves leader cell mitotic success and leader cells provide followers with an escape mechanism (Fig. 8). Interestingly, LCM caused follower cell death and inhibited their colony formation (Figs 6 and 7), suggesting leader cells impact follower cell growth dynamics, perhaps to maintain the leader cell lineage within the greater cellular population. Lastly, how a follower cell secreted factor(s) impacts leader cell growth and mitosis remains an area of interest, where pathways related to growth factor signalling^49^,^50^,^51^ could be candidates for impacting cell survival.

Symbiosis usually involves a mutually beneficial relationship between different organisms^52^; in this case however, the benefit occurs between two phenotypically distinct cellular populations within the collective invasion unit. Symbiosis in cancer has been proposed where cells cooperate to promote survival^53^, especially in the context of heterogeneous subclonal populations (reviewed in ref. 2). Studies in a zebrafish melanoma model show that heterogeneous tumour cell populations cooperate to drive melanoma progression^54^. Similarly, in a mouse xenograft model, inter-clonal cooperation occurs where tumour growth is driven by a minor cell subpopulation^55^, and in breast cancer mouse models inter-clonal cooperation can be essential for Wnt-driven tumours^56^. Taken together, these data suggest that therapeutic approaches aiming to disrupt the symbiotic ecosystem within the tumour cell community could potentially combat the dynamic evolutionary architecture of cancer.

## Methods

### Cell lines and transfections

H1299 and H1792 human NSCLC cells (ATCC, Manassas, VA) were cultured in Roswell Park Memorial Institute (RPMI-1640) media supplemented with 10% foetal bovine serum and 100 units ml^−1^ of penicillin/streptomycin, and maintained at 37 °C and 5% CO_2_. Cell lines were mycoplasma tested and authenticated using single nucleotide polymorphism analysis by the Emory Integrated Genomics Core (see cell line genotyping below).

The gd2PAL-Dendra2 plasmid was obtained from the Gary Bassell lab (Emory University) and was stably transfected into H1299 cells using LT-1 transfection reagent (Mirus), and into H1792 cells using Lipofectamine 2000 (Invitrogen). Geneticin was used to select for Dendra2-expressing cells at 300 μg ml^−1^ concentration (H1299 cells) or 400 μg ml^−1^ (H1792 cells).

To create RFP-leader cells, RFP was subcloned from Lifeact and inserted into the pBabe-puro vector using BamHI and EcoRI. The mCherry-C1 vector was obtained from the Alexa Mattheyses lab (Emory University). mCherry was subcloned into the pBabe-puro vector using Afe1 and EcoR1-HF enzymes. Phoenix-ampho cells were infected as previously described^57^. Puromycin (2 μg ml^−1^; EMD Millipore) was used to select cells expressing the mCherry or RFP plasmid, and expression was verified using immunofluorescence. mCherry-leader cells were made to increase brightness of red signal in mixing experiments.

Oligofectamine (Invitrogen) was used to introduce either two different FN1 siRNAs (Thermo Fisher Scientific) or FAK siRNA into leader cells, and KDR siRNA (Sigma) into H1299 or H1792 parental cells. Cells were treated with siRNA for 48 h, and spheroids were formed after the second day of siRNA treatment and embedded 24–48 h later.

### Cell line genotyping

H1299 samples were processed according to the ABI AmpFLSTR Identifiler PCR Amplification Kit protocol and analysed on the ABI 3130xl Genetic Analyser according to the manufacturer's directions. Amplicons were electrophoresed with the appropriate allelic ladder on the 3130xl Genetic Analyser. Identification analysis was performed using GeneMApper ID software version 3.2.1.

### Reagents and antibodies

Recombinant human VEGFA (R&D, Cat. no. 293-VE) was used for western blotting and live cell imaging experiments to stimulate VEGF signalling. Fibronectin antibody was used for western blotting (1:2,000) and immunofluorescence imaging (1:1,000) (Abcam, Cat. no. ab2413). Notch1 antibody (1:1,000) and VEGF-neutralizing antibody (1:1,000) were obtained from R&D Systems (Cat. nos. AF3647 and MAB293). Primary antibodies against Dll4 (1:500) and VEGFA (1:500) were obtained from Santa Cruz (Cat. nos. sc-365429 and sc-152). VE-cadherin (1:500), GAPDH (1:20,000) and phospho-FAK^Y397^ (1:500) primary antibodies were obtained from Cell Signaling (Cat. nos. 2158, 2118 and 8556). Vimentin antibody was from Sigma (V6630, 1:1,000). E-cadherin and N-cadherin antibodies (1:1,000) were obtained from BD Biosciences (Cat. nos. 610181 and 610920). Alexa Fluor 555 and 647 secondary antibodies (Invitrogen) and DAPI (4′,6-diamidino-2-phenylindole; Invitrogen) were used for 3D immunofluorescence. Horseradish peroxidase-conjugated secondary antibodies (Jackson ImmunoResearch) were used for western blotting.

### Western blot

Cellular protein expression was analysed via western blotting as previously described^57^. To analyse media for secreted proteins, cells were plated in serum-free media for 24 h. Media samples were collected and cell debris eliminated via centrifugation at 300*g* for 5 min. Proteins were precipitated using 100% acetone overnight at −20 °C, then centrifuged at 12,000 rpm for 15 min. Pellets were diluted and boiled in Laemmli sample buffer. Uncropped gels of major data are found in Supplementary Fig. 9.

### Spheroid formation and invasion assays

Spheroids were generated as previously described^57^. Compacted spheroids were collected and resuspended in 2.0 mg ml^−1^ Matrigel (BD Biosciences). Spheroids were plated in a 35 mm glass bottom dish (*In Vitro* Scientific) and incubated at 37 °C overnight. To ensure invasion occurred in 3D and not along the glass bottom, the distance between the spheroid and the dish surface was measured and found to be an average of 76 μm. Images were taken at 0 and 20–24 h post embedding using an Olympus IX51 microscope × 4 (0.13 NA air), × 10 (0.30 NA air) and × 20 (0.45 NA air) with an Infinity2 CCD camera. For drug treatments of spheroids: FAK inhibitor PF-562271 at 2 μM, γ-secretase inhibitor RO4929097 at 10 μM or VEGR2 kinase inhibitor ZM323881 at 10 μM, were added directly to the Matrigel during the embedding process, as well as to the growth media added on top of the matrix.

### Spheroid microscopy

*Fixed cell confocal*. H1299 spheroids were fixed and stained for immunofluorescence as previously described^57^. Spheroids were imaged with the Leica TCS SP8 inverted confocal microscope (× 40 oil HC PL APO, 1.30 NA) using 1.0 μm z-stack intervals and sequential scanning (405 nm DMOD Flexible, 488 nm argon, 514 nm argon).

*Live cell confocal*. H1299 spheroids were embedded in Matrigel and imaged using a Perkin Elmer spinning disk confocal microscope at × 10 (Plan-Neofluar 0.30 NA) mounted onto a Zeiss Axiovert encased at 37 °C with 5% CO_2_. Transmitted light images were acquired every 10 min for 20 h using a Hamamatsu Orca ER CCD camera with 2X2 binning. Quantification of chain dynamics was done using Volocity imaging software. H1299 parental, leader and follower spheroids were imaged using a Leica SP8 inverted confocal microscope with live cell chamber at × 10 (HC Plan Fluotar 0.3 NA). Images were collected every 10 min using a 488 nm argon laser, beginning ∼6 h post embedding for 12 h.

### Spheroid image analysis

4D (*x*,*y*,*z*,*t*) spheroid dynamic images were first projected into 3D (*x*,*y*,*t*) to enhance contrast of dim branches^58^. For each time point (t) and each position in (*x*,*y*) plane, the s.d. of intensity in all *z* direction was calculated^58^. The projected 3D (*x*,*y*,*t*) image sequences were filtered to remove background noise using Matlab function imgaussfilt3. Filtered images were segmented using 3D graph cuts method^59^. The segmented images were polished using Matlab functions imclose and imfill to close gaps and fill holes. The basic morphology features of each spheroid were extracted using Matlab function regionprops. Branch number was quantified using Matlab function bwmorph to generate the skeleton of the spheroid and count the number of skeleton end points. The invasive radius was defined as the distance of the furthest point on the spheroid boundary to the centroid.

Invasive area was quantified by measuring both the total spheroid area around the outer perimeter and the inner spheroid core in ImageJ and taking the difference between the two measures. Spheroid circularity was utilized as an indirect measure of sheet-like invasion, and was quantified in ImageJ by measuring the spheroid outer invasive perimeter.

### SaGA technique

H1299-Dendra2 or H1792-Dendra2 cells were plated for spheroids, embedded in Matrigel and incubated overnight. After ∼16 h of invasion, spheroid plates were imaged using the Nikon A1R live cell laser scanning confocal. Spheroids were imaged using the × 10 objective (0.3 NA DIC) and photoconversion was performed at a 3 × zoom using the A1R software. The 405 nm laser was used to photoconvert cells of interest at laser power 10–30%, which was found to not induce DNA damage in the cells as measured by yH2AX staining in cell nuclei (Supplementary Fig. 2B). To extract photoconverted cells, the protease dispase was faster and gentler on cells when compared to trypsin using FACS analysis (Supplementary Fig. 2C). Therefore, the Matrigel matrix was degraded and single cell suspension was achieved using dispase I at 1 μg ml^−1^ with intermittent manual disruption via pipetting. The protease activity was inactivated using media and samples were centrifuged. Upon resuspension, the samples were analysed via FACS for TexasRed and FITC expression (Supplementary Fig. 2D). To improve signal:noise for FACS cell isolation, photoconversion was optimized by varying the 405 nm light excitation scan iterations, while considering cell viability post photoconversion. Low photoconversion efficiency was defined as a red fluorescence signal <300 a.f.u. and high efficiency as >300 a.f.u. (Supplementary Fig. 2E,F). Both photoconverted (red) and non-photoconverted (green) populations were isolated from the cell sorter with two levels of gate stringency and imaged with fluorescence microscopy post-sorting to assess purity of red-positive cells (P2 population; Supplementary Fig. 2G). In the low-efficiency condition, low- and high-gate stringency resulted in a contaminated (non-photoconverted cells present) P2 population (Supplementary Fig. 2G, left). Using high-efficiency photoconversion and a high-gate stringency gave nearly a 100% pure P2 population (Supplementary Fig. 2G, right) and this approach was continued throughout. For purified leader or follower cell collections, 30–50 cells were sorted per well and expanded. For microarray analysis, 10 cells per well in triplicate were collected (see below).

### Microarray transcriptome studies

Cells were processed using the Ovation One-Direct System and Encore Biotin Module (NuGEN Technologies, Inc., San Carlos, CA). Biotin-labelled cDNA was hybridized to the Affymetrix Human Gene ST 2.0 gene expression microarray and further processed on the GeneChip Instrument System for Array Cartridges (Affymetrix, Santa Clara, CA). All steps were carried out according to the manufacturer's protocol. Briefly, RNA from 1–20 cells was reverse transcribed using a proprietary RNA/DNA duplexed primer. ss-cDNA was converted to ds-cDNA and linearly amplified in a single primer isothermal amplification reaction. Amplified cDNA was then fragmented and labelled. Biotin-labelled cDNA was hybridized to the Human Gene ST 2.0 GeneChip at 45° C for 40 h. Hybridized microarrays were washed and stained on the Affymetrix GeneChip 450 fluidics station using the appropriate chip-dependent fluidics script. Arrays were scanned and intensity data extracted using the Affymetrix 7G scanner and the Command Console software suite.

*Microarray analysis*. The raw CEL files for all 12 samples were GC content adjusted, RMA background corrected, log2 transformed, quantile normalized and mean probset summarized using Partek Genomics Suite v6.6 (PGS; Partek Inc., St Louis, MO)^60^. The probesets were annotated with the Affymetrix Human Gene 2.0 ST annotation file and expression values represented at the gene level.

The impact of photoconversion on gene expression was controlled by comparing invasive cells that have been photoconverted (IR) to invasive cells that were not photoconverted (NG). Any gene that significantly changed between these two conditions was denoted as alterations due to the photoconversion process and was excluded from analysis. Relative to these controls, significant expression differences between leader and follower cells were tested with the four following hypotheses: (1) mean gene expression differences between leader versus follower cells is greater than mean (absolute) expression differences between control (IR and NG) cells; (2) mean expression differences between leader versus follower cells is greater than zero; (3) mean gene expression differences between followers versus leaders is greater than mean (absolute) expression differences between control (IR and NG) cells; and (4) mean expression differences between followers versus leaders is greater than zero. Hypothesis testing was done based on 500 permutations. For each permutation of the data, a *t*-statistic was defined by taking all pairs of differences among samples. In specific, nine pairs of expression differences between leaders and followers were formed by taking one sample from each of leader and follower cells. Likewise, nine pairs of expression differences between the two defined controls were formed. Using these nine difference pairs, both a two- and one-sample *t*-statistic were defined for each gene for testing respective hypotheses 1 and 2; the differences were reversed for testing hypotheses 3 and 4. By permuting the expression difference pairs between groups (leaders and followers differences versus controls differences), a *P* value was estimated for each gene based on comparing the number of times the permuted data exceeded both the one- and two-sample *t*-statistic formed based on the observed data. Genes with *P*<0.05 were selected as the differentially expressed genes in the leaders (*N*=788) and followers (*N*=634). The heat map of differentially expressed genes was generated using heat map.2R function. The biological pathways enriched among these differentially upregulated genes were searched against several curated databases using the functional interaction networks in Reactome FI Cytoscape plugin^61^,^62^.

### Proliferation assays and mitosis analysis

For the proliferation assays, H1299 leader and follower cells were plated in triplicate in a 24-well plate. At days 1–3, cells were counted using an automatic cell counter (BioRad). For mitotic event analyses, H1299 RFP-leader and follower cells were plated in an eight-well LabTek glass bottom slide either alone or in co-culture. After 6 h, cells were imaged every 5 min for 21 h on the Leica SP8 inverted confocal microscope at × 10 using a 488 nm argon laser. Mitotic events were analysed from these images using Leica Application Suite X software. The length of time from prophase to anaphase and anaphase to cytokinesis was determined by morphological features. The beginning of prophase was defined as the first image where the cell became spherical and increased Dendra2 fluorescence, the beginning of anaphase was defined as the first image where the chromosomes were visibly separated and the cell has begun elongating, and cytokinesis was defined as the first image where the two daughter cells are separated by a plasma membrane. The presence of a variety of mitotic defects was defined by morphological abnormalities. Cytokinetic instability was defined as what appears to be initially >two daughter cells with excessive membrane blebbing and cell shape deformation during cytokinesis, but over time is corrected to two daughter cells. Cell death events were counted based on morphological changes consistent with cell death phenotypes (loss of all cell motility and membrane dynamics, shrinkage of cell, nuclear fragmentation, formation of apoptotic bodies, phagocytosis by neighbouring cells and so on).

### Cell cycle analysis

H1299 follower and leader cells were plated in 100 mm tissue culture dishes. After 24 h, cells were washed and fresh RMPI-1640 media supplemented with 0 or 10% FBS was added to the cells. After 20 h, cells were collected and fixed in 95% ethanol at −20 °C. Cells were stored at 4 °C for 24 h before staining with DNA staining buffer (4 μg ml^−1^ DAPI, 0.25% Triton-X 100 in 1X PBS). DAPI expression was analysed by flow cytometry on a BD FACSCanto-II cytometer using FACSDiva software. FlowJo software was used to exclude doublets and determine the distribution of cells within G0/G1, S and G2 peaks.

### Colony formation assays

H1299 parental, follower and leader cells, or H1792 follower and leader cells, were plated in 35 mm tissue culture dishes at 500 cells per plate. Cells were grown for 2 weeks, and media (RPMI-1640, 24-h follower conditioned media, or 24-h leader conditioned media) was refreshed every 3 days. To create conditioned media, 10 × 10^4^ leader cells or 7.2 × 10^4^ cells were seeded in a six-well plate so as to reach ∼70% confluence. After 24 h, cells were washed twice with 1 × PBS and then 1.5 ml of RPMI-1640 without FBS was added to each well. After another 24 h, media was centrifuged to remove cells and debris, and 24-h conditioned media was added to colony formation assays. After 2 weeks, colony formation assays were stained with crystal violet (6% glutaraldehyde, 0.5% crystal violet in 1 × PBS) for 30 min before rinsing thoroughly with water. Colony surface area and the number of colonies with more than 50 cells were quantified using Fiji imaging software (ImageJ).

### Force sensor experiments

Potassium phosphate monobasic (≥99.0%) and (3-aminopropyl) trimethoxysilane (97%, APTMS) were purchased from Sigma-Aldrich (St Louis, MO). Fluorescent dye Alexa647 DIBO alkyne was purchased from Life Technologies (Grand Island, NY). 4-Azido-L-phenylalanine was purchased from Chem-Impex International (Wood Dale, IL). Ni-NTA Agarose (#30210) was purchased from Qiagen (Valencia, CA) P2 gel size exclusion beads were purchased from BioRad (Hercules, CA). Succinimidyl PEG NHS 2,000 Da and Lipoic acid PEG NHS 3,400 Da were purchased from Nanocs (Boston, Ma). Gold nanoparticles were purchased from nanoComposix (San Diego, CA).

I27-based construct was designed with N-terminal ligand TVYAVTGRGDSPASSAA and two C-terminal cysteines for immobilization onto AuNPs. The pET22b plasmid encoding an I27-based sensor with a TAG codon was co-transformed with pEVOL-pAzF plasmid into electrocompetent BL21(DE3) *E. coli* cells. Cells were grown at 37 °C in the presence of ampicillin, chloramphenicol and 0.2% glucose to an optical density (OD) of 0.2, at which 1 mM of 4-azido-L-phenylalanine was added. At an OD of 0.4, L-arabinose was added to a final concentration of 0.02% (w/v) and at an OD of 0.8, isopropyl β-D-1-thiogalactopyranoside was added to a final concentration of 1 mM. Cells were shaken for 16 h at 30 °C, purified by Ni^2+^ affinity chromatography and stored at −80 °C in 0.1 M potassium phosphate buffer (pH 7.4).

I27-based protein sensor was incubated with DIBO-A647 for 1 h at 37 °C, followed by incubation at room temperature for 24 h. The sensor was next purified using P2 gel size exclusion beads and the labelling ratio was quantified by UV–vis absorption (NanoDrop).

Glass coverslips were piranha etched for 30 min, functionalized with an APTMS solution in acetone for 1 h and thermally annealed at 80 °C for 20 min. Subsequently, the surfaces were passivated with 5% (w/v) mPEG-NHS and 0.5% (w/v) lipoic acid PEG NHS in 0.1 M fresh sodium bicarbonate overnight at 4 °C. After passivation, 12 nM of AuNPs (diameter=9 nm) were incubated onto the surface for 20 min.

### Statistical analysis

A two-tailed unpaired Student's *t*-tests were used to analyse statistical significance between two conditions in an experiment. For experiments with three or more comparisons, an ordinary one-way ANOVA with a Tukey's multiple comparisons test was used. Significance was assigned to *P* values <0.05. Error bars represent the mean±s.e.m.

### Data availability

Microarray data that support the findings of this study have been deposited in GEO archive with accession code GSE93865. All other remaining data are available within the article and Supplementary Files, or available from the authors on request.

## Additional information

**How to cite this article:** Konen, J. *et al*. Image-guided genomics of phenotypically heterogeneous populations reveals vascular signalling during symbiotic collective cancer invasion. *Nat. Commun.* **8,** 15078 doi: 10.1038/ncomms15078 (2017).

**Publisher's note:** Springer Nature remains neutral with regard to jurisdictional claims in published maps and institutional affiliations.

## Supplementary Material

Supplementary Information Supplementary Figures

Supplementary Movie 1 H1299 spheroids show collective invasion in 3-D spheroid invasion assays. H1299 NSCLC spheroids were embedded in a 3-D Matrigel matrix and imaged over time using live cell microscopy.

Supplementary Movie 2 H1792 spheroids show collective invasion in 3-D spheroid invasion assay. H1792 NSCLC spheroids were embedded in Matrigel. Images were acquired after spheroids were allowed to invade for ~18 hours.

Supplementary Movie 3 H1299 purified leader cells maintain their invasiveness in 3-D spheroid invasion assays. Purified H1299 leader cell spheroids were embedded in a 3-D Matrigel matrix. After ~8 hours, spheroids were imaged on the live cell confocal microscope over time.

Supplementary Movie 4 H1299 purified follower cells show limited invasion in 3-D spheroid invasion assays. H1299 follower cell spheroids were imaged over 8 hours using live cell microscopy.

Supplementary Movie 5 H1299 follower cells display dynamic lamellipodia in 2-D motility but limited net movement. H1299 follower cells were plated in 2-D. Six hours after plating, to allow cells time to adhere to the dish, cells were imaged every 10 minutes using live cell confocal microscopy.

Supplementary Movie 6 The addition of leader cells promotes follower cell motility in 2-D. H1299 follower cells were plated in co-culture with RFP-leader cells. Both populations express Dendra2 and thus are green, whereas only leader cells are RFP-positive.
