# Supplementary material for: Image-guided genomics of phenotypically heterogeneous populations reveals vascular signalling during symbiotic collective cancer invasion
Source: Nat Commun. 2017 May 12;8:15078. doi: 10.1038/ncomms15078 (PMC5437311; doi:10.1038/ncomms15078)
Supplement: Supplementary Information — Supplementary Figures [file ncomms15078-s1.pdf]

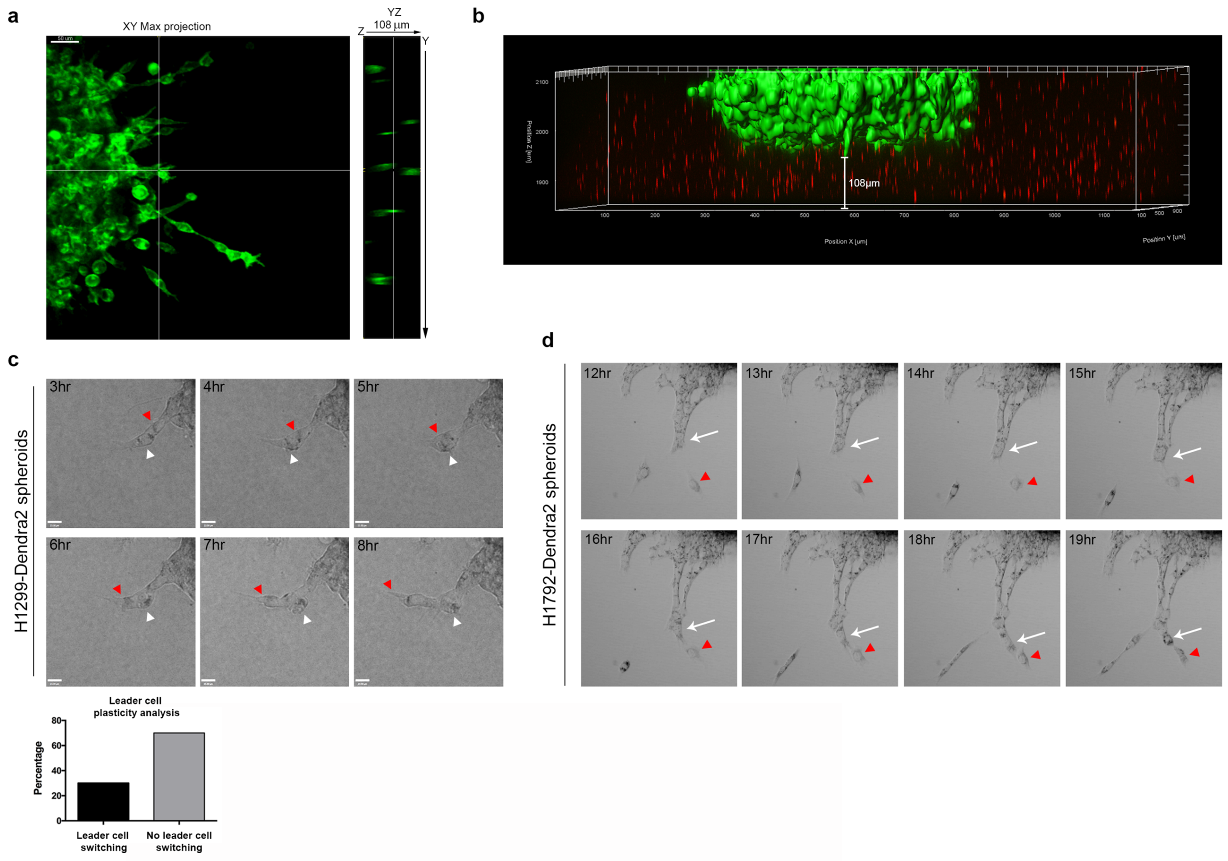

Supplementary Figure 1. H1299 and H1792 spheroids show collective invasion in a 3-D Matrigel matrix. (**a**) H1299-Dendra spheroid with multiple collective invasion chains. YZ projection on right shows that invasion occurs in multiple Z-planes. (**b**) 3-D image of a H1299-Dendra2 spheroid embedded within Matrigel mixed with red fluorescent beads to show invasion occurs in a 3-D context. The bottom half of the spheroid and matrix are shown, and the distance between the spheroid and dish surface is marked. (**c**) H1299-Dendra2 spheroid time lapse showing a single invasive chain with leader cell switching. White arrowhead = original leader cell. Red arrowhead = new leader cell. (**d**) H1792-Dendra spheroids were embedded in Matrigel and imaged using live cell confocal microscopy. Images were acquired every ten minutes. Representative still images are shown, taken every 1 hour. Red arrowhead = leader cell. White arrow = follower cell within the invasive chain.

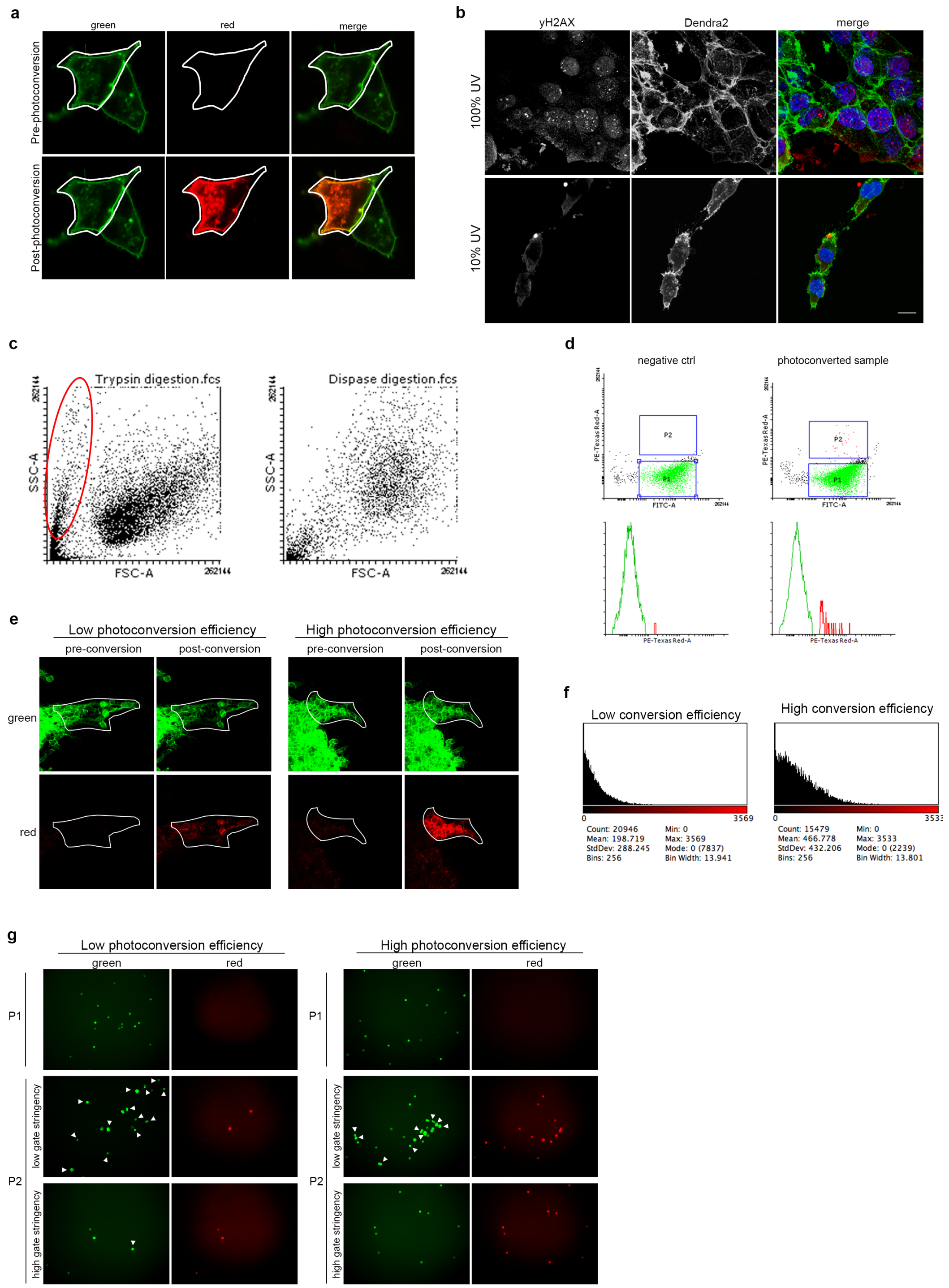

Supplementary Figure 2. Development and optimization of the SaGA technique. (**a**) H1299-Dendra2 cells in a 2-D monolayer were photoconverted using the 405nm laser. White outline around left cell shows the region of interest for photoconversion. (**b**) Immunofluorescence imaging of γH2AX in H1299-Dendra2 spheroids. Invading cells were exposed to either 100% or 10% 405nm laser power, which is comparable to the laser power utilized during photoconversion. (**c**) H1299-Dendra2 spheroids embedded within a Matrigel matrix were degraded using either trypsin or dispase. Single cell suspensions were then analyzed via FACS and forward and side scatter plots are shown. Large cellular debris fraction is marked in the red circle. (**d**) Photoconverted cells were analyzed via FACS using TexasRed to detect red positive cells. Gate was set using a negative control (H1299-Dendra2 cells that were not photoconverted). P1 = non-photoconverted cells, P2 = photoconverted cells. Histograms of Texas Red expression in each sample is shown below. (**e**) Examples of low photoconversion efficiency (<300 a.f.u.) and high photoconversion efficiency (>300 a.f.u.) for optimization of sorting. (**f**) Histogram of post-photoconversion images in **e**. Histograms represent only the red channel intensity. (**g**) Using low (left) or high (right) photoconversion efficiency settings from **e**, photoconverted cells (P2) were collected on two different

gate stringencies on the FACS machine. P1 denotes non-photoconverted cells as a negative control. Images were taken immediately after sorting to identify the sorting purity. Arrowheads = contaminating non-photoconverted cells.

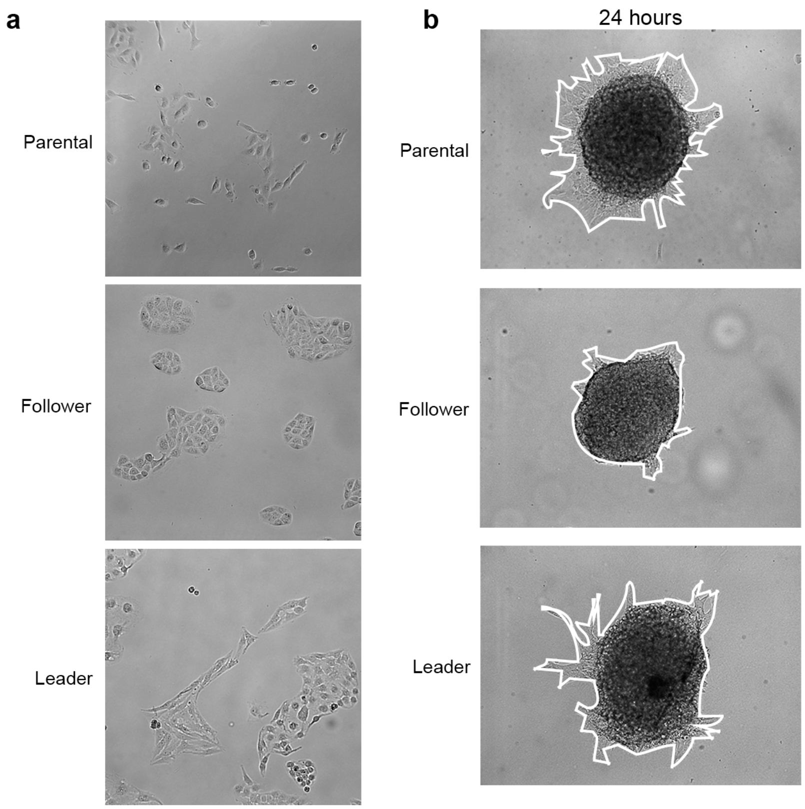

Supplementary Figure 3. H1792-Dendra2 leader and follower cells show different morphology in 2-D culture and invasive patterning in 3-D spheroids. (**a**) Brightfield images were taken of H1792-Dendra2 parental, follower, and leader cells in 2-D culture. (**b**) Spheroid invasion assay with H1792-Dendra2 parental, follower, and leader spheroids. White outline shows invasive area.

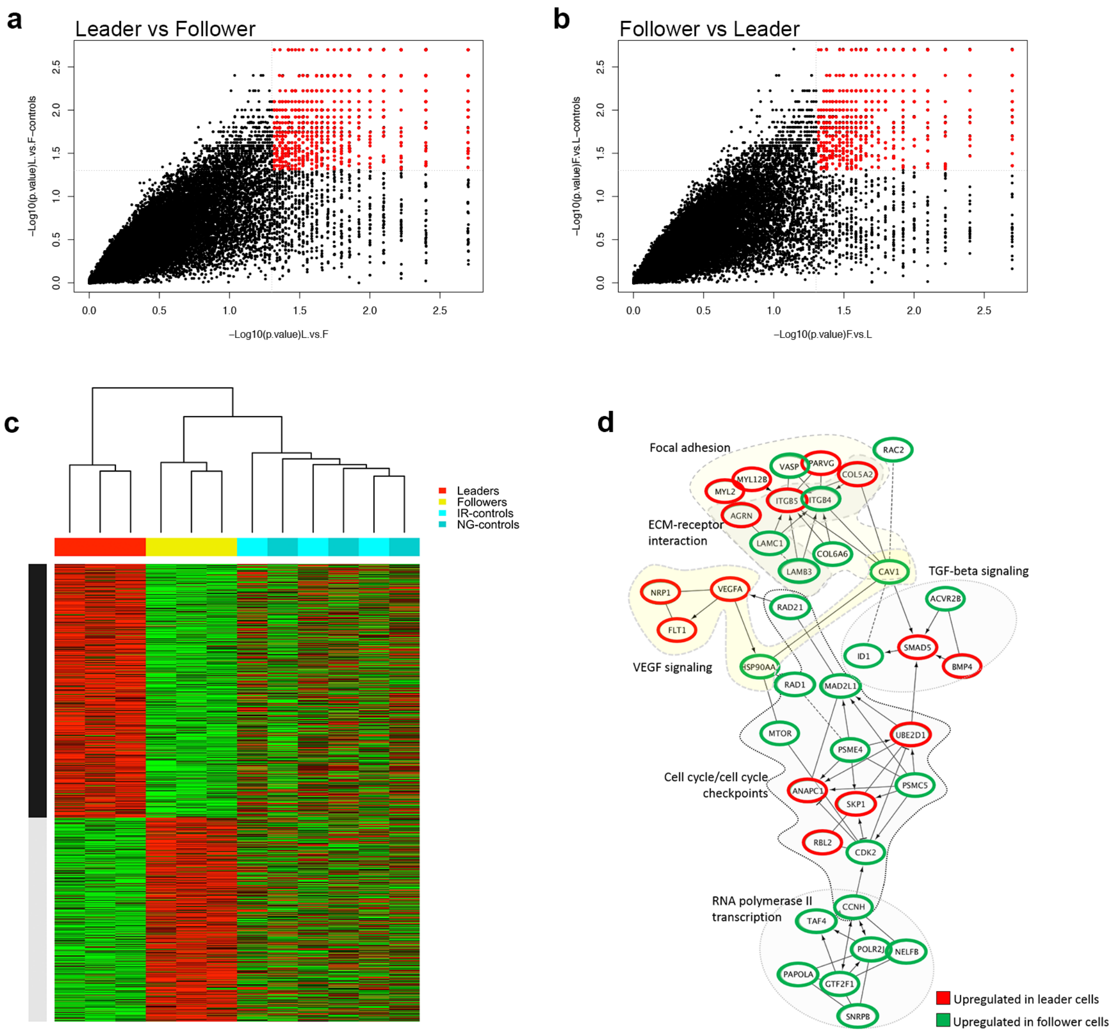

Supplementary Figure 4. Gene expression analysis of purified leader and follower cells. (**a**) Scatter plot showing permutation results of (-log10) p-values based on testing mean (pairwise among samples) expression differences greater in leaders (L) versus followers (F) (x-axis), and testing mean (pairwise among samples) expression differences between leaders and follows greater than differences in controls (y-axis). Each dot represents a gene. Genes whose mean expression was significantly (p.value £ 0.05) greater based on testing both hypotheses are highlighted in red. (**b**) Analysis from **a** was repeated for expression differences greater in followers (F) versus leaders (L). (**c**) Unsupervised hierarchical clustering of 1422 significantly differentially expressed genes in leader (red; N=788) and follower (yellow; N=634) cells compared to controls (IR and NG; cyan shades). The hierarchical clustering was performed using Pearson correlation distance measure and average linkage method. The results represented are gene level expression log2 transformed after quantile normalization. (**d**) Reactome FI network of significantly over expressed genes in the leader and follower cells relative to controls. Each node (or circle) represents a gene. Colors in the network indicate the over-expressed genes in the leader (red) and followers (green), respectively. The direct experimental and predicted interactions between the genes are shown by solid and dashed black lines, respectively. The known activating/catalyzing and inactivating (or inhibiting) interactions are shown by arrow-headed ("->") and bar-headed lines ("-|"), respectively. Only the GO processes with more than three genes are shown. The network is created using Reactome FI network tool[63] in Cytoscape[62]. Some of the pathways highlighted in the network are signaling by RNA polymerase II transcription, TGF-beta, focal adhesion, ECM-receptor interaction, cell cycle/cell cycle checkpoints, and VEGF signaling pathway.

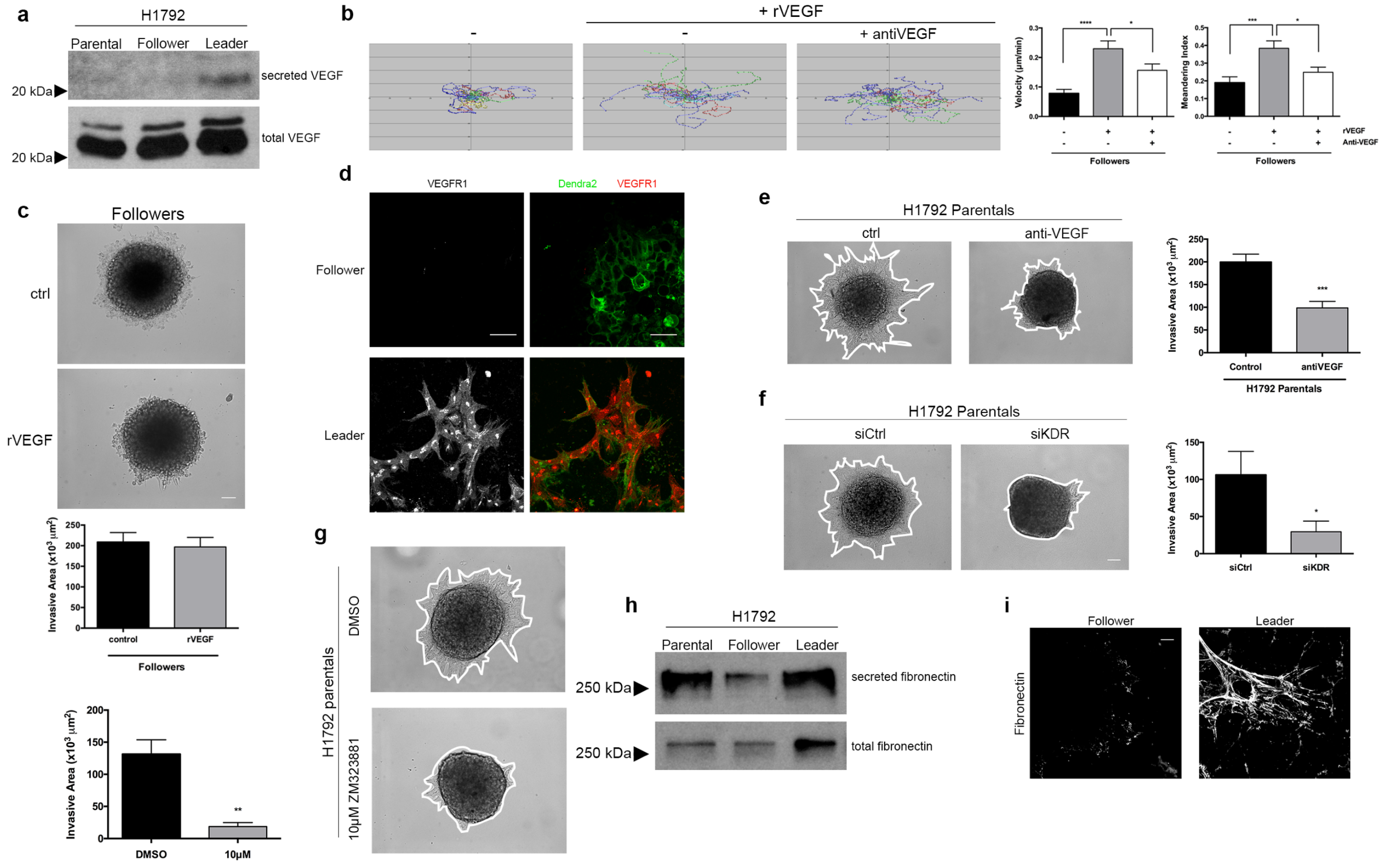

Supplementary Figure 5. Leader cells upregulate VEGF and fibronectin secretion as compared to follower cells. (**a**) Western blot showing expression of VEGF in media samples (secreted) and whole cell lysates from H1792 parental, follower, and leader cells. (**b**) Recombinant human VEGFA (rVEGF) was added to follower cells and their motility tracked in 2-D over time. Cell track plots are shown, and quantification of velocity and meandering index are graphed to the right. n = 21 cells. *p<0.05, ***p<0.001, using an ordinary one-way ANOVA. (**c**) Spheroid invasion assay of follower cells with the addition of rVEGF. Spheroid invasive area is graphed below. n = 10 spheroids. Scale bar = 100 µm. (**d**) Immunofluorescence imaging of VEGFR1 in H1792 follower and leader spheroids. Scale bar = 50µm. (**e**) H1792 parental cells were treated with VEGF-neutralizing antibody (anti-VEGF) or vehicle control. Invasive area was measured and graphed below. n = 6 spheroids. **p<0.01 using a Student's *t*-test. (**f**) H1792 parental spheroids were analyzed for invasion after knockdown of VEGFR2 (*KDR*) via targeted siRNA. Scrambled siRNA was used as a negative control. Quantification of invasive area is shown to the right. n = 4-5 spheroids. *p<0.05 using a Student's *t*-test. (**g**) H1792 parental spheroids were treated with VEGFR2 kinase inhibitor, ZM323881 at 10µM, and invasion was compared to vehicle control (DMSO). A graph of invasive area is shown to the left. n = 5 spheroids. **p<0.01. (**h**) Expression of fibronectin in both cell lysates and media (secreted) samples collected from H1792 parental, follower, and leader cells. (**i**) Immunofluorescence imaging of extracellular fibronectin in H1792 follower and leader spheroids. Scale bar = 20µm.

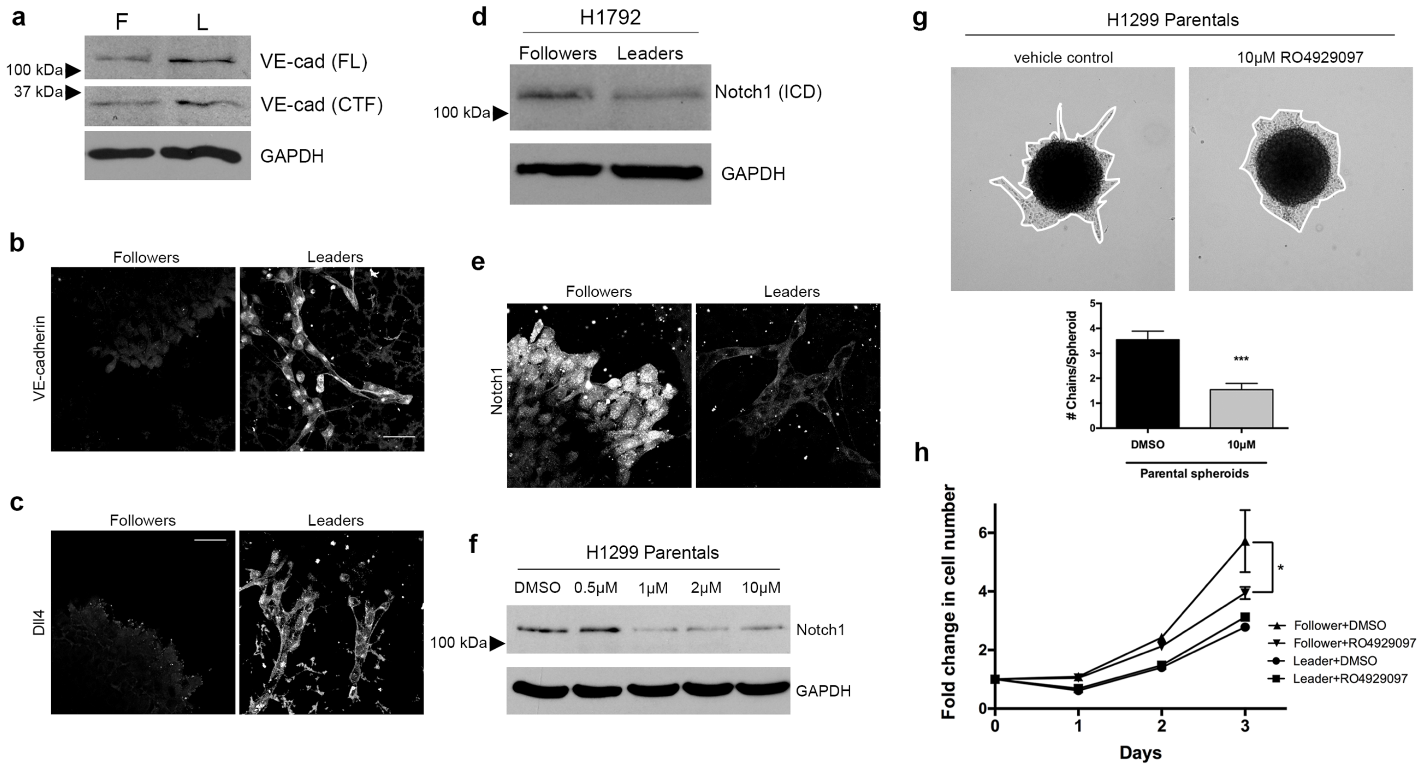

Supplementary Figure 6. Leader cells mimic expression profiles seen during tip-stalk cell vasculogenesis. (**a**) Western blot showing VE-cadherin expression, both full length and the C-terminal fragment, in follower and leader cell lysates. GAPDH was used as a loading control. (**b**) Immunofluorescence imaging of VE-cadherin in follower and leader cell spheroids. Scale = 50μm. (**c**) Immunofluorescence imaging of Dll4 in follower and leader spheroids. Scale bar = 50μm. (**d**) Western blot showing Notch1 intracellular domain (ICD) expression in H1792 follower and leader cells. (**e**) Immunofluorescence imaging of Notch1 in follower and leader spheroids. Scale = 50μm. (**f**) Western blot showing Notch1 intracellular domain expression in H1299 parental cells. Cells were treated with increasing doses of the γ-secretase inhibitor, RO4929097, or DMSO control. GAPDH was used as a loading control. (**g**) H1299 parental spheroids were embedded in Matrigel in the presence of 10μM of RO4929097 or vehicle control. The number of invasive chains per spheroid was quantified. n = 11 spheroids. ***p<0.001 using a Student's *t*-test. (**h**) H1299 follower and leader cells were plated in 2-D with 10μM RO4929097 γ-secretase inhibitor and counted every day for 3 days. Graph shows fold change in total cell number over time. n = 3. Error bars represent SEM. *p<0.05 using multiple t-tests with Holm-Sidak method to correct for multiple comparisons.

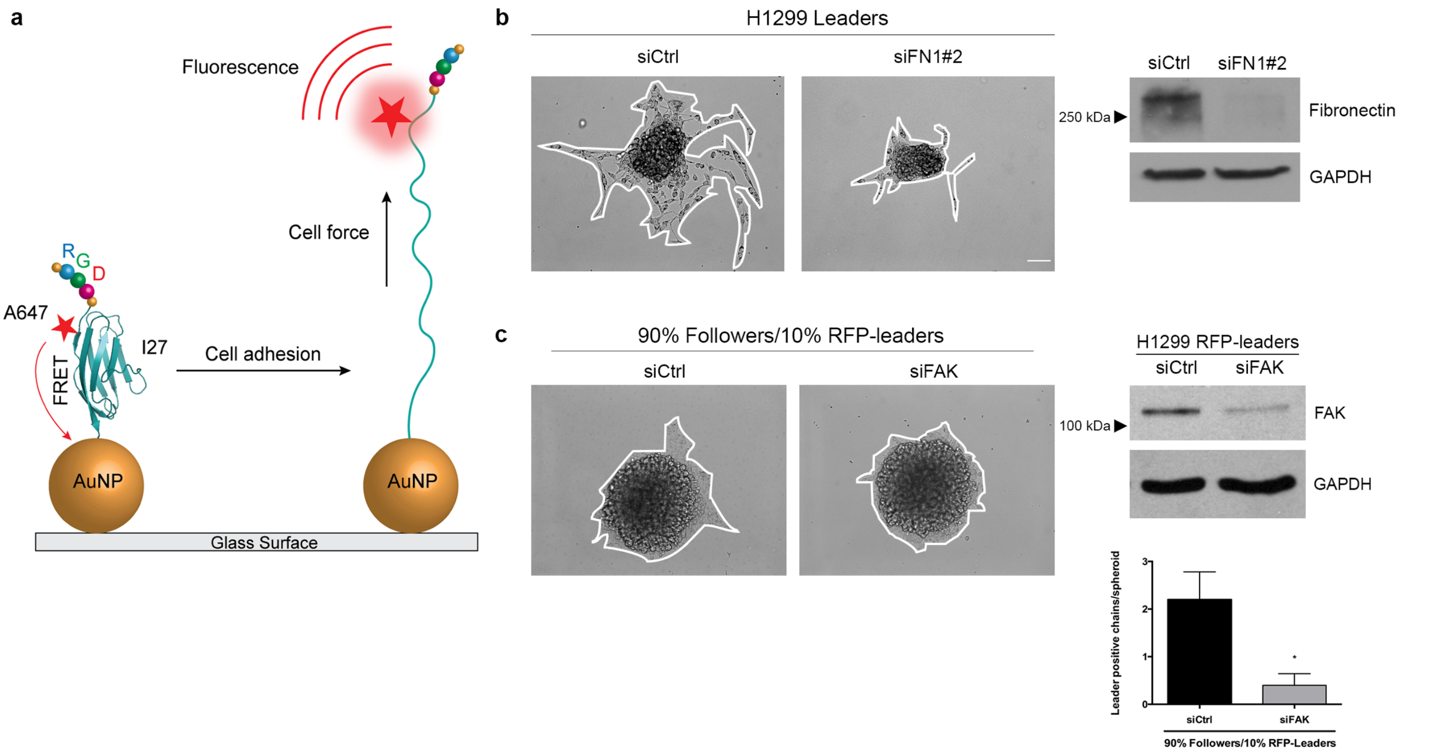

Supplementary Figure 7. Adhesion studies in leader cells. (**a**) Tension probe utilized to study adhesion forces (PDB ID: 1TIT). The experimental details used to prepare the probes and their response function is reported in [33]. Briefly, the tension sensor is comprised of an engineered protein modified at the N-terminus with RGD integrin binding motif and with two cysteines at the C terminus for immobilization onto a 9 nm gold particle. Alexa647 organic dye was site-specifically conjugated to a non-canonical amino acid (p-azidophenylalanine) that was engineered near the N-terminus and allowed for copper free click chemistry. In the resting state, the titin-based probe is folded and the dye is quenched by the gold nanoparticle. When integrin receptors recognize the RGD sequence and unfolds the protein, this leads to separation of the dye from the gold nanoparticle. This results in dequenching of the dye, and a significant enhancement of the fluorescence signal at the sites of the integrin adhesions. Importantly, the tension fluorescence indicates a force magnitude that exceeds 36 pN. (**b**) H1299 leader spheroids were formed from cells depleted of fibronectin (*FN1*) via a second targeted siRNA. Scrambled siRNA was used as a negative control. Western blot confirming knockdown is shown to the right. (**c**) FAK siRNA or scrambled siRNA control was introduced into H1299 RFP-leader cells. Western confirming knockdown is shown to the right. Leader cells were plated as mixed spheroids at a 90:10 ratio with H1299 follower cells. RFP-leader positive chains were calculated and the graph is shown in the lower right. n = 5 spheroids. *p<0.05 using a Student's *t*-test.

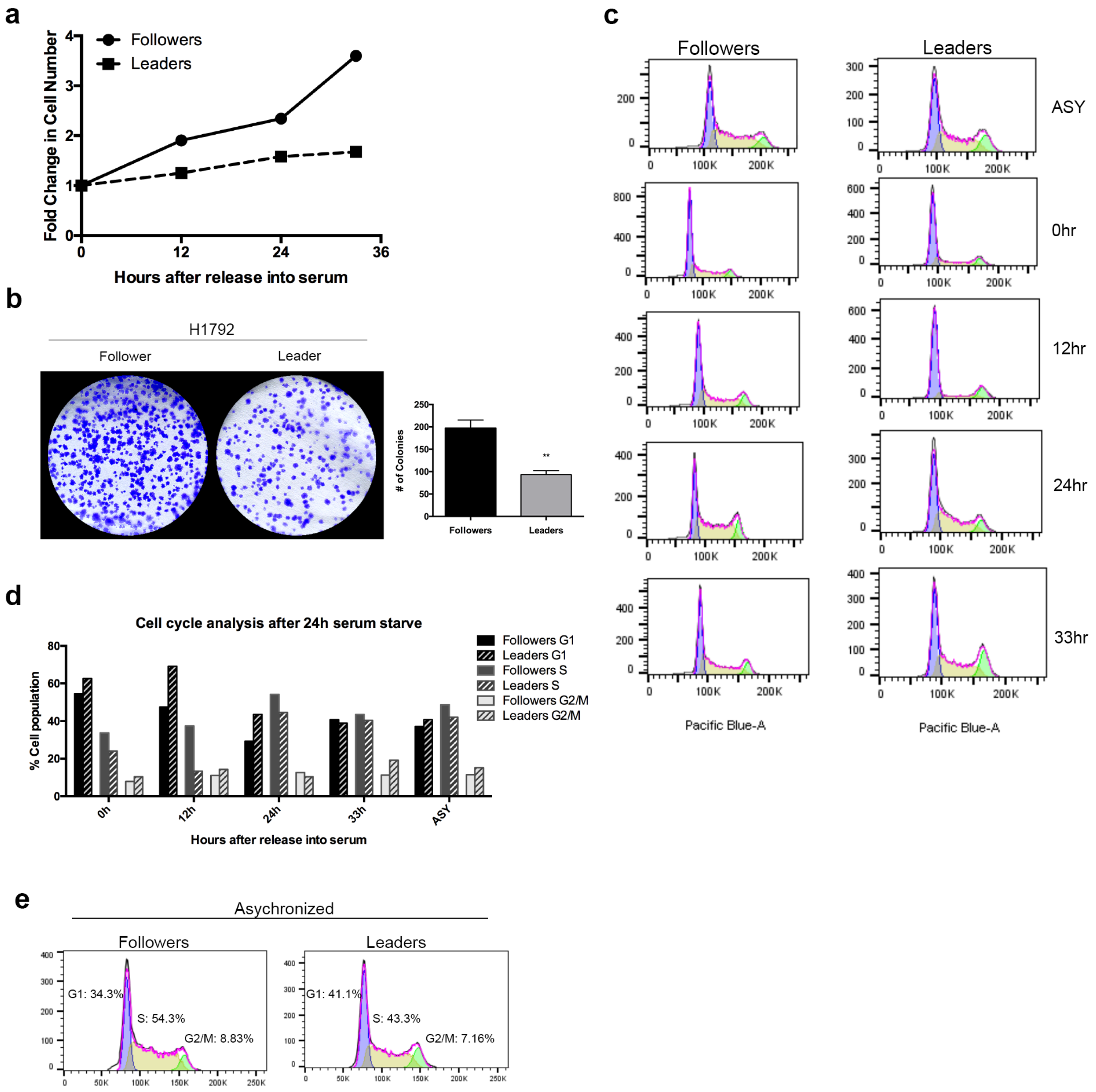

Supplementary Figure 8. Cell cycle analysis of follower and leader cells. (**a**) A sample of H1299 follower and leader cells were collected prior to fixation for the cell cycle analysis and counted using an automatic cell counting machine. Fold change in total cell number is shown over the time course. (**b**) H1792 follower and leader cells were plated for a colony formation assay and stained with crystal violet. Quantification of the number of colonies is shown to the right. n = 3. **p<0.01 using a Student's *t*-test. (**c,d**) H1299 follower and leader cells were serum starved for 24 hours, then released by the addition of normal growth media for various lengths of time. Cells were collected for both cell growth (**a**) and cell cycle analysis. (**c**) Cells were fixed at various times after release from serum starvation and stained with DAPI for cell cycle analysis. (**d**) Quantification of each cell cycle phase from **c** in follower and leader cells. (**e**) H1299 follower and leader cells were fixed and stained with DAPI for cell cycle analysis using flow cytometry. Cells were not first synchronized before fixation.

Fig 4C

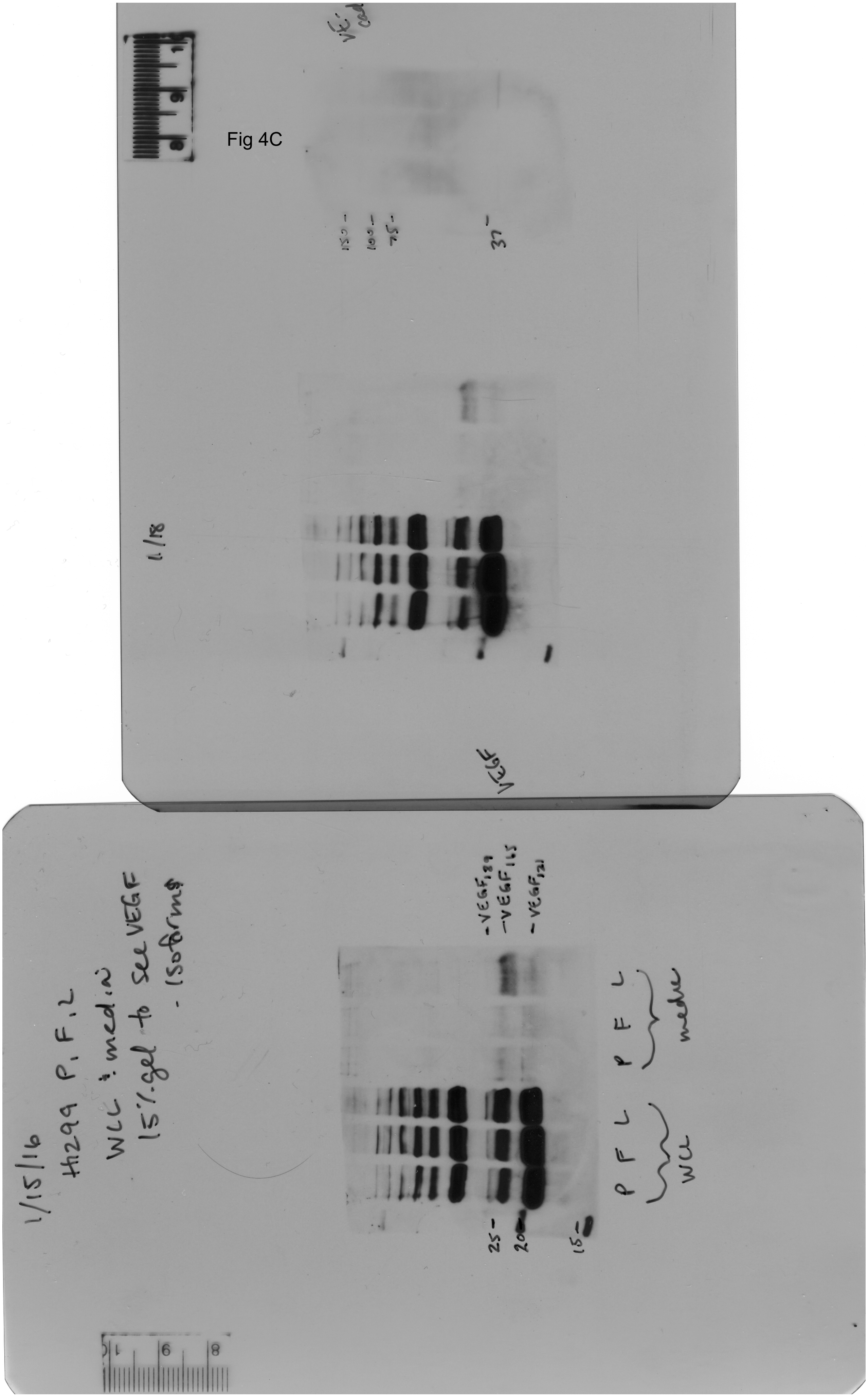
150 -
100 -
75 -
37 -
1/18
VEGF
1/15/16
H1299 P, F, L
WCL & media
15% gel to see VEGF
- isoforms
-VEGF189
-VEGF165
-VEGF121
25 -
20 -
15 -
P F L
WCL
P F L
media

Supp Fig 5a

H1792 parental, leader and follower cells

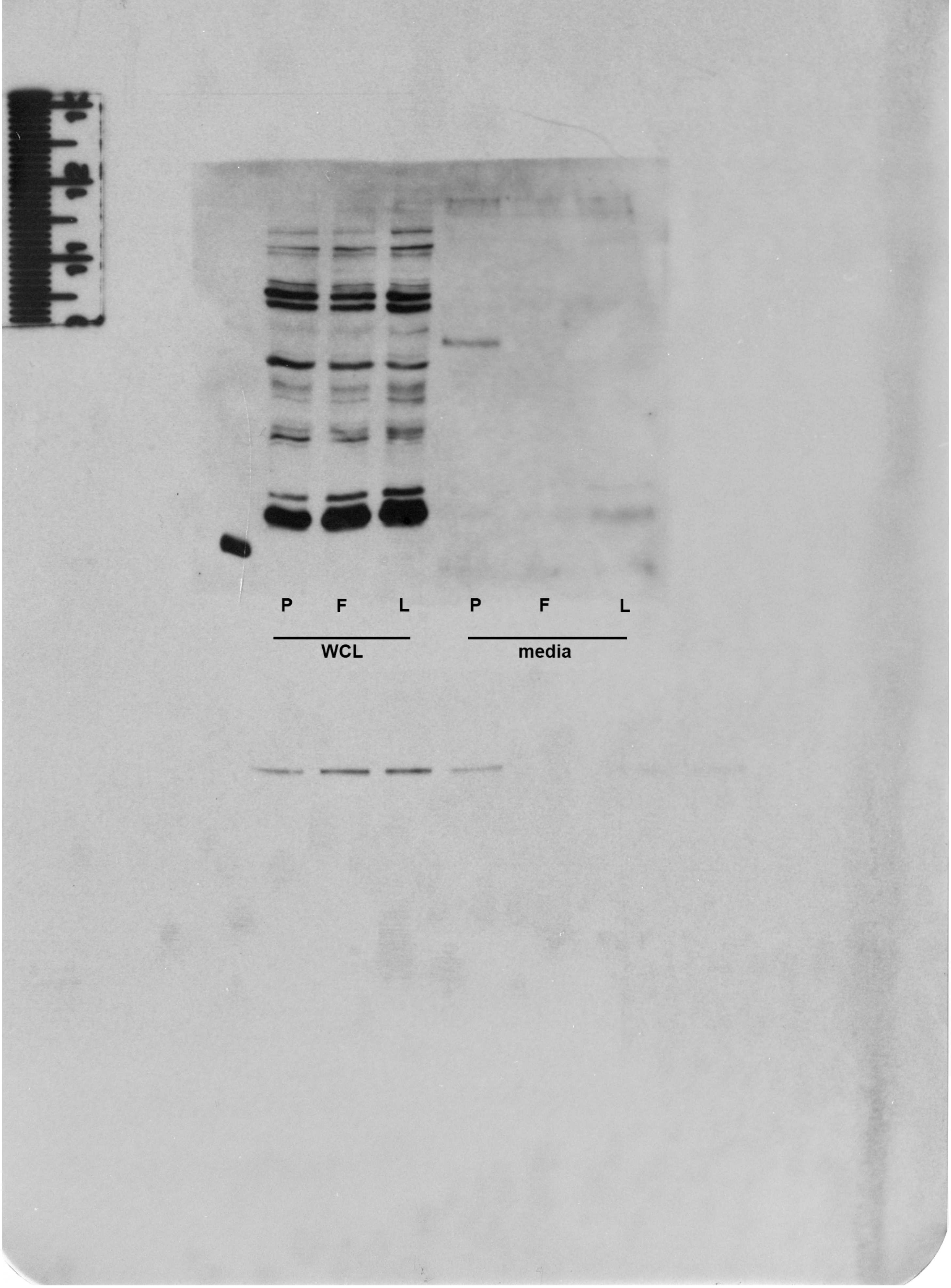

Supp Fig 5h

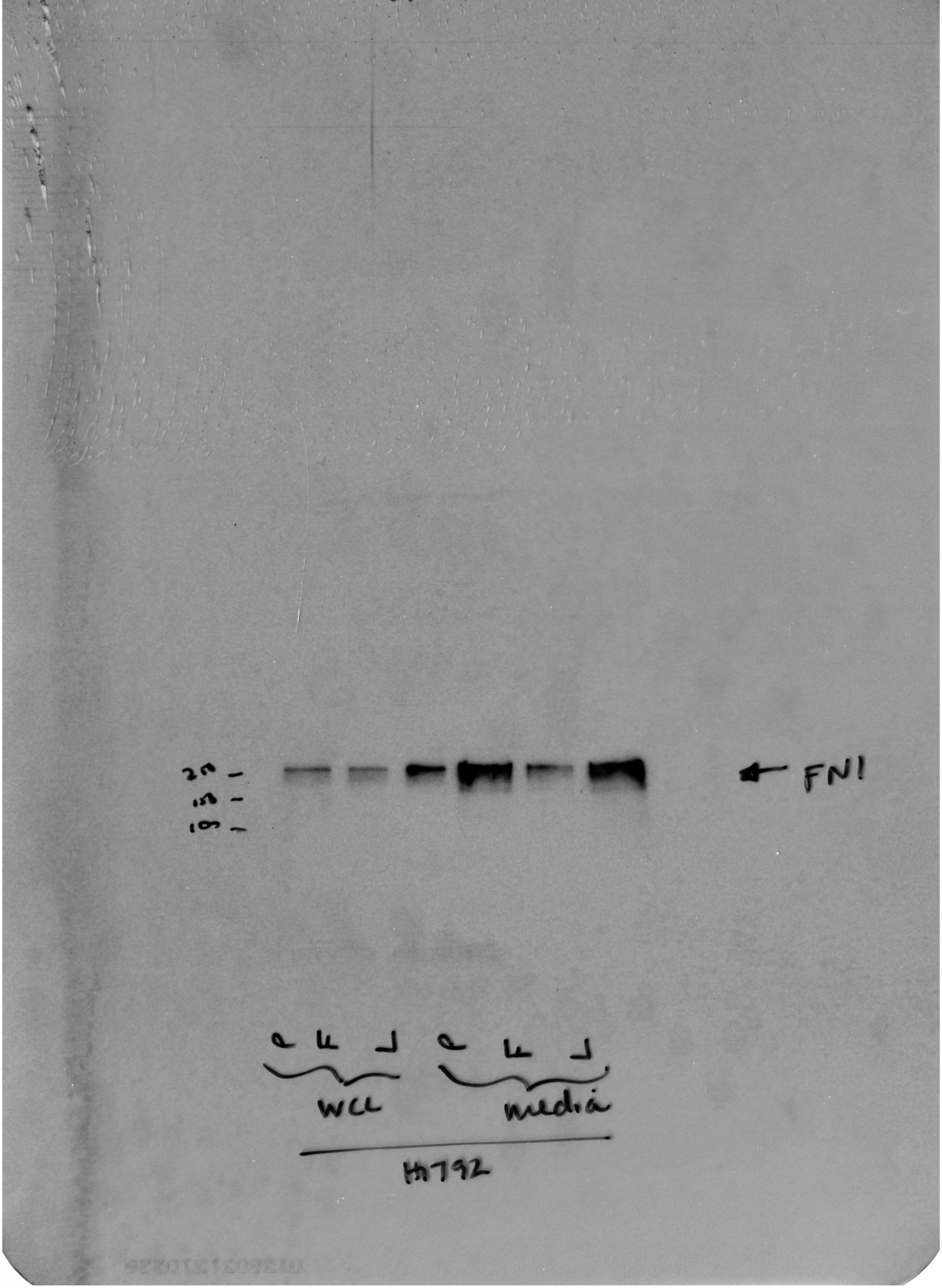

Supp Fig 6a

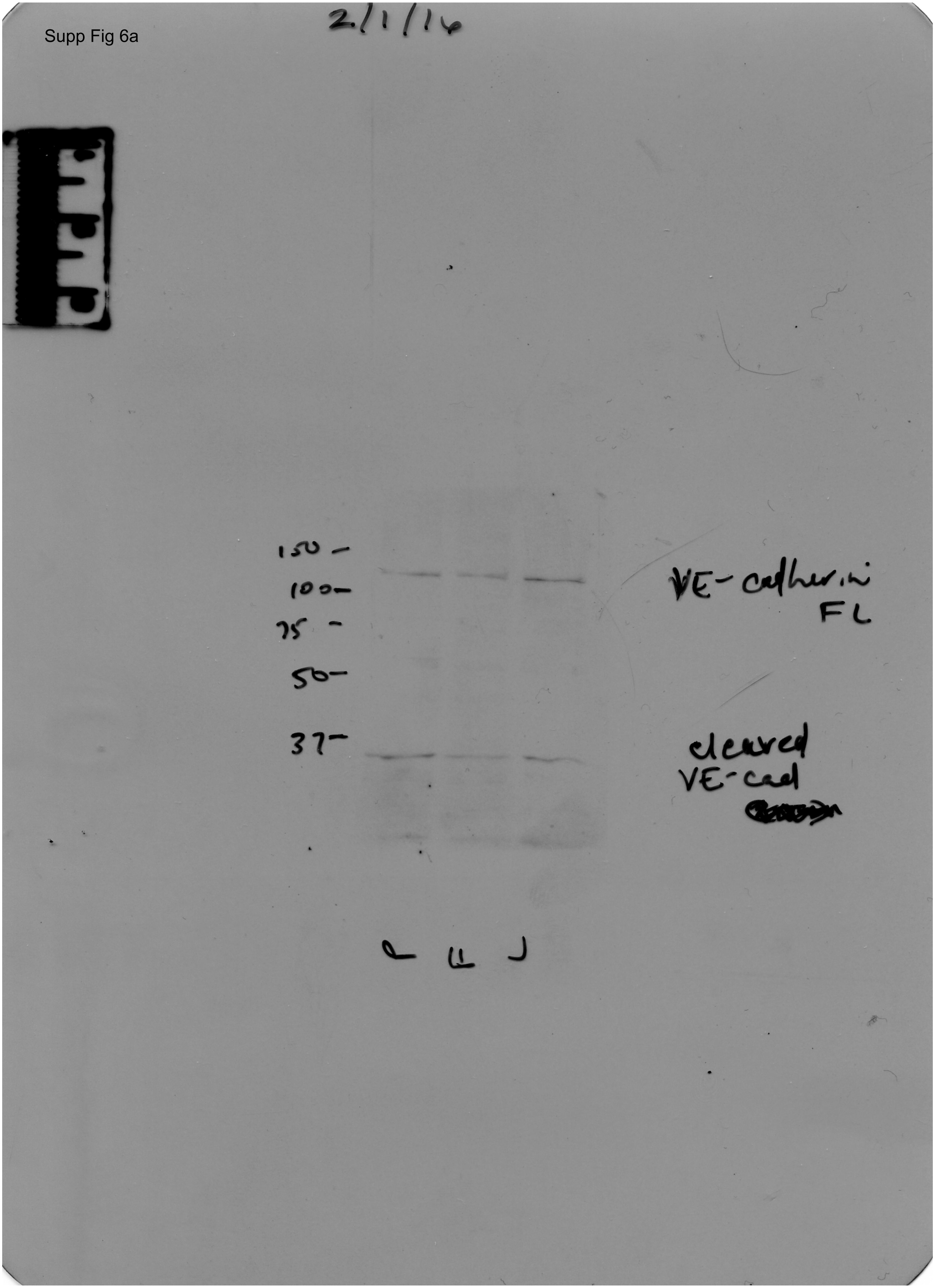

Supp Fig 6d

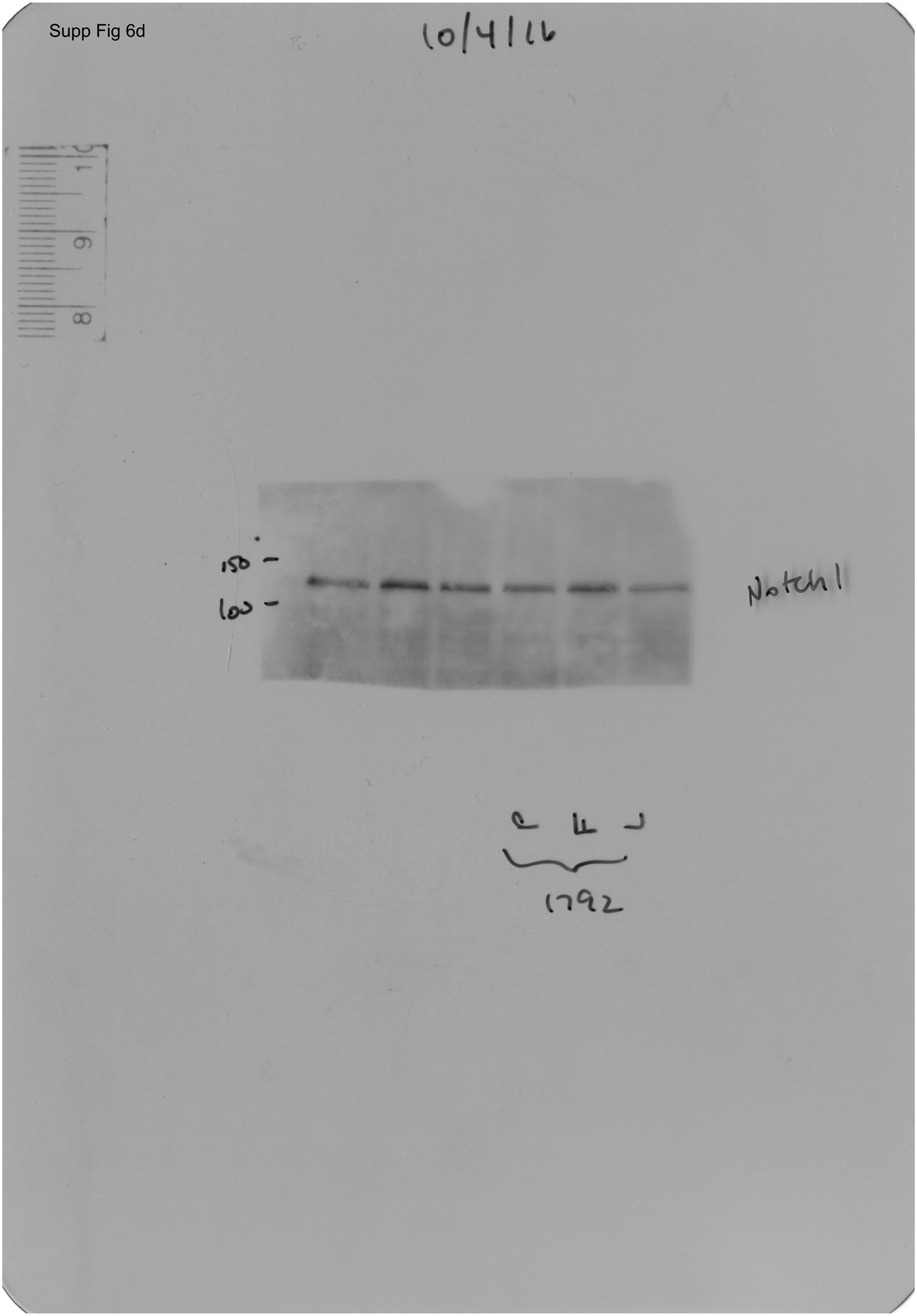

Supp Fig 6f

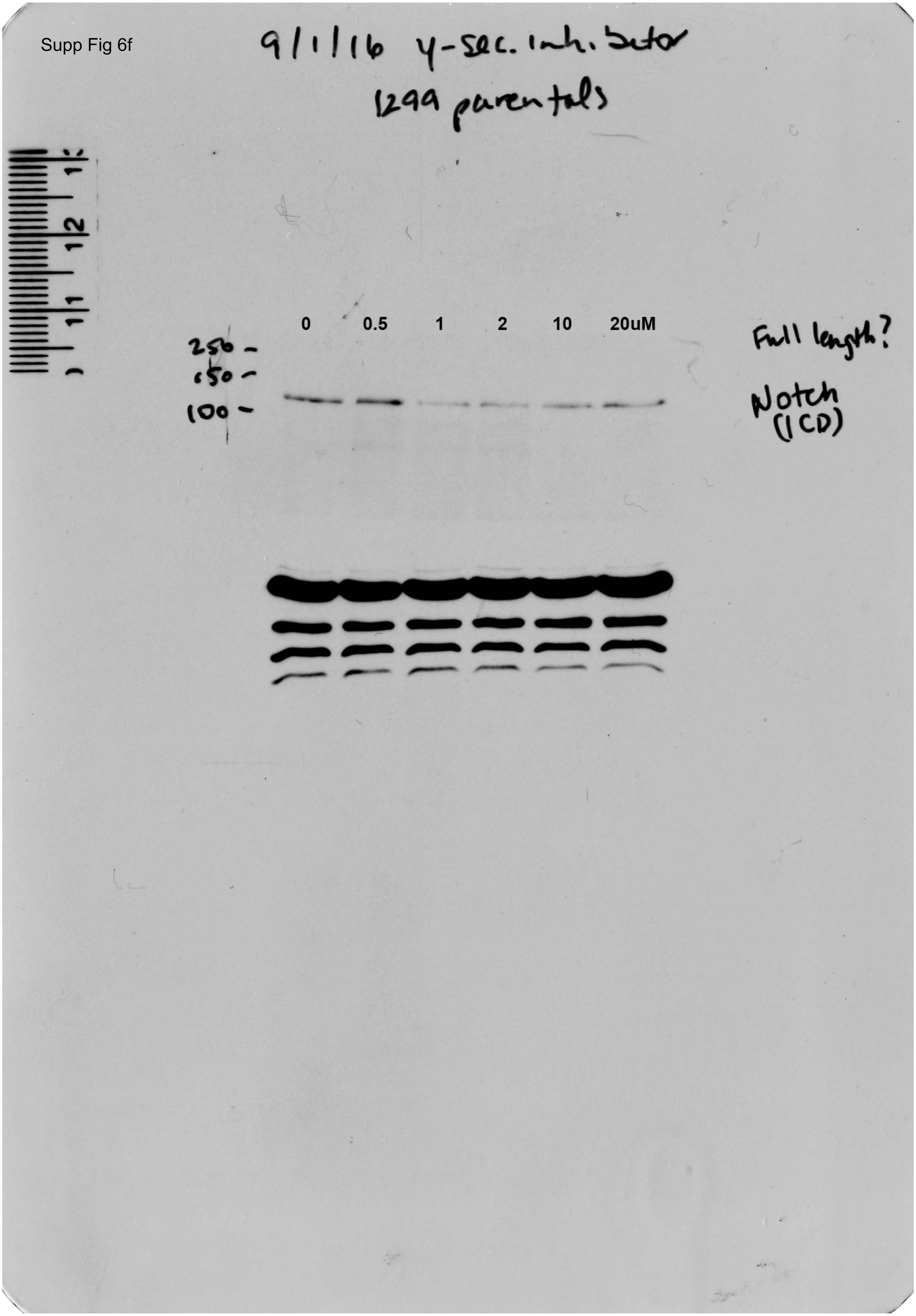
9/1/16 γ-sec. inhibitor
1299 parentals
0
0.5
1
2
10
20uM
250 -
150 -
100 -
Full length?
Notch (ICD)

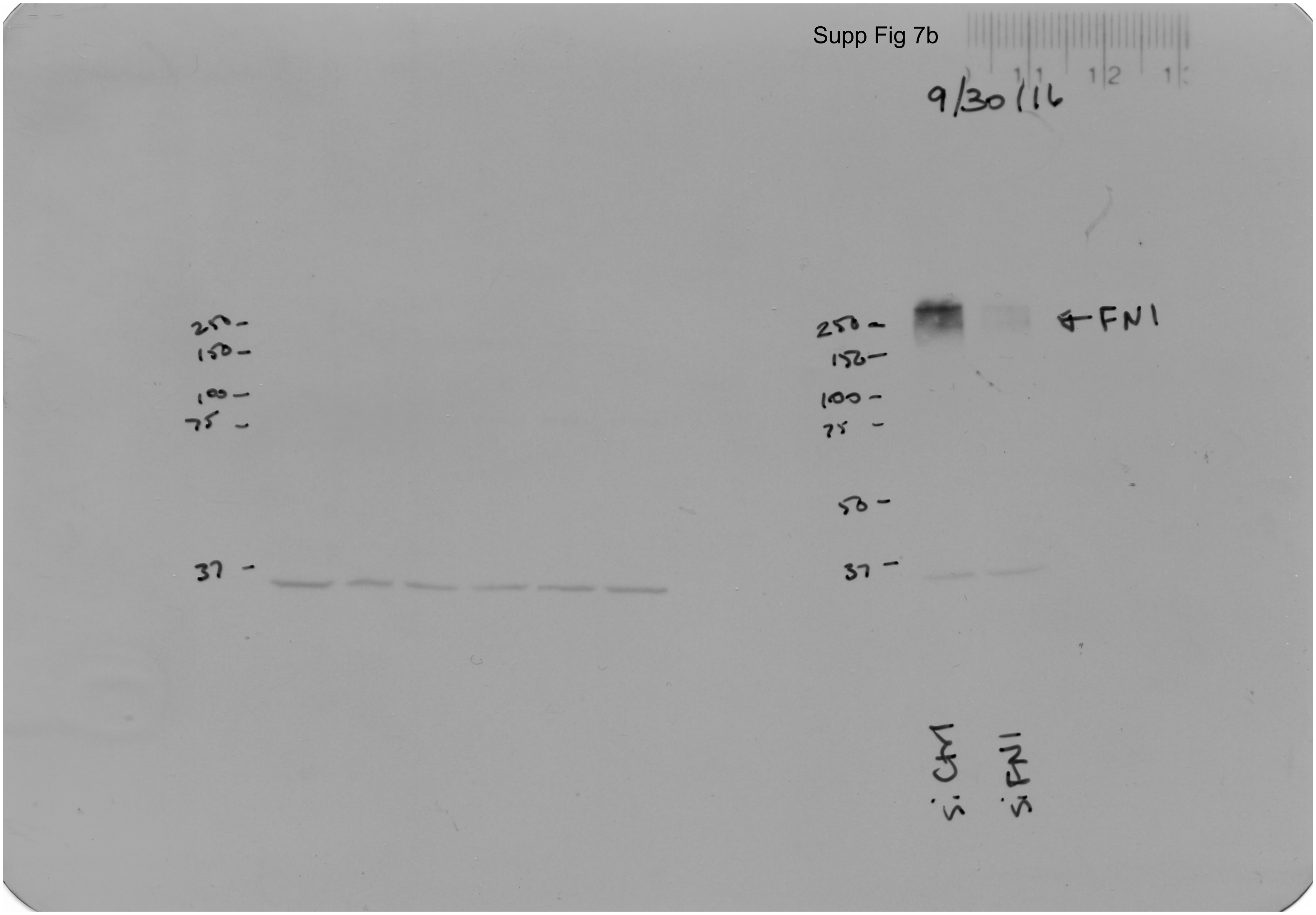
Supp Fig 7b
9/30/16
250
150
100
75
37
250
150
100
75
50
37
FN1
si Ctrl
si FN1

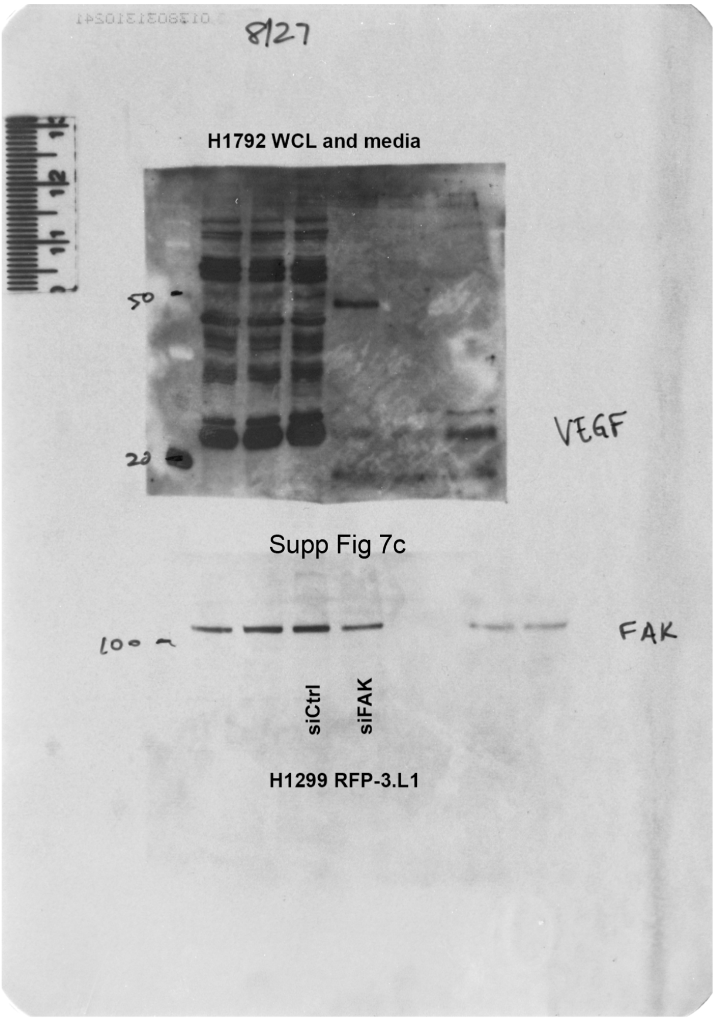

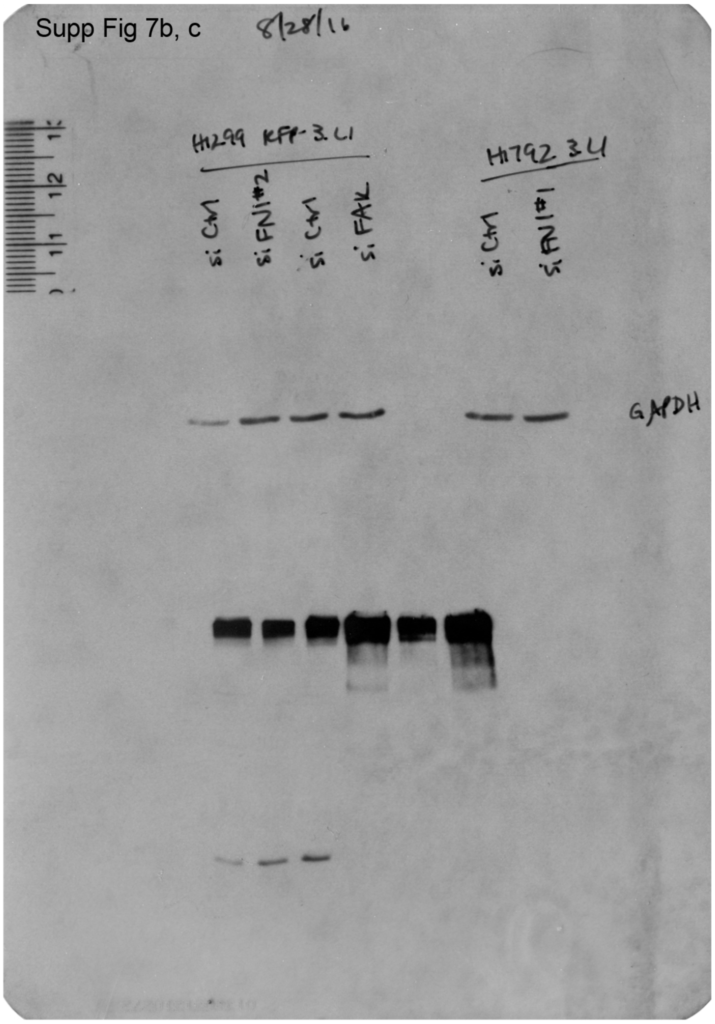

Supplementary Figure 9. Uncropped western blots.

External ID	TH01		D21S11		D5S818		D13S317		D7S820		D16S539		CSF1PO		AMEL		vWA		TPOX	
2800M Control DNA	6	9.3	29	31.2	12	12	9	11	8	11	9	13	12	12	X	Y	16	19	11	11
H1299 Follower#1	6	9.3	32.2	32.2	11	11	12	12	10	10	12	13	12	12	X	X	16	18	8	8
H1299 Follower#2	6	9.3	32.2	32.2	11	11	12	12	10	10	12	13	12	12	X	X	16	18	8	8
H1299 Follower#3	6	9.3	32.2	32.2	11	11	12	12	10	10	12	13	12	12	X	X	16	18	8	8
H1299 Leader#1	6	9.3	32.2	32.2	11	11	12	12	10	10	12	13	12	12	X	X	16	18	8	8
H1299 Leader#2	6	9.3	32.2	32.2	11	11	12	12	10	10	12	13	12	12	X	X	16	18	8	8
H1299-Dendra2 parent	6	9.3	32.2	32.2	11	11	12	12	10	10	12	13	12	12	X	X	16	18	8	8

Supplementary Table 1. SNP analysis of H1299 leader and follower purified clones. H1299-Dendra2 parental cells collected and submitted for SNP analysis, and compared to the two leader and three follower purified clones. Identification analysis was performed using GeneMApper ID software.
